# mRNA and miRNA profiling of Zika virus-infected human umbilical cord mesenchymal stem cells identifies miR-142-5p as an antiviral factor

**DOI:** 10.1080/22221751.2020.1821581

**Published:** 2020-09-22

**Authors:** Rak-Kyun Seong, Jae Kyung Lee, Geum Joon Cho, Mukesh Kumar, Ok Sarah Shin

**Affiliations:** a Department of Biomedical Sciences, College of Medicine, Korea University Guro Hospital, Seoul, Republic of Korea; b Department of Obstetrics and Gynaecology, College of Medicine, Korea University Guro Hospital, Seoul, Republic of Korea; c Department of Biology, Georgia State University, Atlanta, Georgia, USA

**Keywords:** ZIKV, hUCMSCs, miRNA, innate immunity, RNA-seq, small RNA-seq

## Abstract

Zika virus (ZIKV) infection during pregnancy is associated with congenital brain abnormalities, a finding that highlights the urgent need to understand mother-to-fetus transmission mechanisms. Human umbilical cord mesenchymal stem cells (hUCMSCs) are susceptible to ZIKV infection but the underlying mechanisms of viral susceptibility remain largely unexplored. In this study, we have characterized and compared host mRNA and miRNA expression profiles in hUCMSCs after infection with two lineages of ZIKV, African (MR766) and Asian (PRVABC59). RNA sequencing analysis identified differentially expressed genes involved in anti-viral immunity and mitochondrial dynamics following ZIKV infection. In particular, ZIKV-infected hUCMSCs displayed mitochondrial elongation and the treatment of hUCMSCs with mitochondrial fission inhibitor led to a dose-dependent increase in ZIKV gene expression and decrease in anti-viral signalling pathways. Moreover, small RNA sequencing analysis identified several significantly up- or down-regulated microRNAs. Interestingly, miR-142-5p was significantly downregulated upon ZIKV infection, whereas cellular targets of miR-142-5p, *IL6ST* and *ITGAV*, were upregulated. Overexpression of miR-142-5p resulted in the suppression of ZIKV replication. Furthermore, blocking *ITGAV* expression resulted in a significant suppression of ZIKV binding to cells, suggesting a potential role of ITGAV in ZIKV entry. In conclusion, these results demonstrate both common and specific host responses to African and Asian ZIKV lineages and indicate miR-142-5p as a key regulator of ZIKV replication in the umbilical cords.

## Introduction

Zika virus (ZIKV) is a mosquito-borne single-stranded RNA virus, which has been largely neglected for more than 60 years since its first isolation in 1947 [1]. The rapid expansion of ZIKV in South and Central America in 2015 has gained medical attention emphasizing the capacity of ZIKV to spread to non-endemic regions [2, 3]. Although ZIKV infection is usually asymptomatic and self-limiting in non-pregnant individuals, ZIKV infection during pregnancy has been demonstrated to cause fetal developmental abnormalities and pregnancy loss [4–6]. ZIKV replication has been detected in the placenta, amniotic fluid, cerebrospinal fluid and fetal brain tissue [4, 7–9]. To date, effective antiviral agents or vaccines to treat or prevent ZIKV-associated disease are not available for human use. Although it is proposed that ZIKV pathogenesis is associated with its broad cell and tissue tropism during the first trimester pregnancy [10, 11], the detailed mechanisms of how ZIKV crosses the physical barrier of the placenta are yet to be fully determined.

Placenta is essential in host defence against viral infections, and its protective functions range from a physical barrier of the multinuclear syncytium to the innate and adaptive immune responses [12, 13]. The placenta, which is connected to the fetus by the umbilical cord, provides a rich source of mesenchymal stromal or stem cells (MSCs) that are multipotent, self-renewable, and able to differentiate *in vitro* to bone, cartilage and adipose tissues [14]. A recent study by Tipnis *et al* suggested that umbilical cord-derived MSCs (hUCMSCs) possess essential immunomodulatory phenotypes mediated by the production of soluble factors such as tumour growth factor–β (TGF-β), interleukin-10 (IL-10) and other unidentified factors [15]. Additionally, El Costa *et al* provides evidence using *ex vivo* model that Asian lineage ZIKV is able to infect and damage the tissue architecture of maternal decidua basalis, fetal placenta and umbilical cord [10]. Although these studies show that the umbilical cord and hUCMSCs, in particular, were highly susceptible to ZIKV infection, the role and contribution of hUCMSCs in ZIKV infection remains to be determined.

MicroRNAs (miRNAs) are non-coding RNAs that are 20–25 nucleotides long. miRNAs have an important role in regulating stem cell self-renewal and differentiation by suppressing the translation of selected mRNAs [16]. During viral infections, miRNAs can be used either by viruses to promote virus replication, or by host cells to alter differential gene expression in response to viral infection [17]. Small RNA-sequencing represents a useful method to study genome-wide miRNA profiles [18], whereas high-throughput RNA-seq technology provides a fast, integrative and effective way to reveal the dynamic changes in host gene expression during virus infection [19]. Little is currently known about the role of miRNAs and miRNA-regulated networks in ZIKV-induced pathogenesis.

In this study, we used next generation sequencing technology to characterize and compare host mRNA and miRNA expression profiles in hUCMSCs infected with two lineages of ZIKV. ZIKV-induced mitochondrial dysfunction was observed by transmission electron microscopy, confocal microscopy, and mitochondrial flux analysis. Furthermore, we analysed the role of selected mRNA and miRNAs in ZIKV replication.

## Materials and methods

### Cells and viruses

Umbilical cords were collected from healthy donors and mesenchymal stem cells were isolated from the umbilical cords using an in-house established protocol. All methods were carried out in accordance with relevant Institutional Review Board (IRB) guidelines and regulations (Korea University Guro Hospital, Seoul, Korea). In brief, the cord was cleaned with phosphate-buffered saline (Invitrogen, Carlsbad, CA, USA), blood clots were removed, and the cord was dissected into smaller explants and placed on tissue culture dishes in Dulbecco’s modified Eagle’s medium supplemented with 10% fetal bovine serum (FBS) (Corning Mediatech) and 1% non-essential amino acids (Sigma, St Louis, MO, USA). The cells were allowed to grow out from the explants, expand into monolayers and supplemented with fresh media every other day. hUCMSCs from passage numbers 3–10 were used for all the experiments.

A549 adenocarcinoma cells, JAR choriocarcinoma cells, Hela cells and Vero African green monkey kidney epithelial cells were obtained from the American Type Culture Collection (Manassas, VA, USA). A549 and JAR cells were cultured in RPMI supplemented with 10% FBS and antibiotics, whereas Hela and Vero cells were cultured in DMEM supplemented with 5% FBS and antibiotics. Human neural progenitor cells (hNPCs) were generated as previously described [20–22].

ZIKV MR766 (African lineage) and PRVABC59 (Asian lineage) strains were purchased from ATCC and propagated in Vero cells. Viral titers were determined using a standard plaque assay as described previously [20, 21]. For the measurement of 50% tissue culture infective dose (TCID50), supernatants from ZIKV-infected hUCMSCs were collected at 24, 48, and 72 hpi. TCID50 was determined using the Spearman-Kärber method and expressed as TCID50 units/mL.

### Confocal microscopy

Cells seeded onto coverslips in 24-well plates were infected with PBS (Mock) or ZIKV. Cells were washed with PBS, fixed with 4% paraformaldehyde and permeabilized with 0.1% Triton X-100. MitoTRACKER Green was purchased from Thermo Fisher Scientific, whereas CD73 antibody was obtained from eBioscience. Cells were then stained with anti-pan-flavivirus envelope (E) monoclonal antibody (1:200 dilution; Abcam, Cambridge, UK), followed by anti-mouse Alexa 594 conjugated antibody (1:200 dilution, Invitrogen). Coverslips were mounted on glass slides using mounting media containing 4,6-diamidino-2-phenylindole (DAPI) and were examined using a confocal microscope (LSM700; Carl Zeiss, Oberkochen, Germany).

### RNA sequencing and bioinformatics analysis

Total RNA was isolated using Direct-zol RNA mini Prep Kit (Zymo Research, Germany) following manufacturer’s instructions. RNA sequencing was performed using an Illumina HiSeq 2500 sequencer (Illumina, San Diego, CA) as previously described [18, 23, 24]. Sequence reads were aligned to the reference genome (Gene bank accession no. hg19) using Tophat (v 1.4.1, Baltimore, MD, USA). The raw RNA-Seq data files were deposited in NCBI’s Gene Expression Omnibus (GEO) and are accessible via GEO Series accession number GSE122193.

The gene expression level was calculated based on fragments per kilobase of exon per million mapped reads using Cufflinks v2.1.13 from Ensembl release 72. Functional classification of the genes was performed for gene ontology (GO) in Database for Annotation, Visualization and Integrated Discovery (DAVID). The GO database classifies genes according to the three categories of biological process, cellular component, and molecular function, and predicts the function of the selected genes. Pathway analysis was performed using Kyoto Encyclopaedia of Genes and Genomes (KEGG), allowing the visualization of metabolic pathways and molecular interaction networks captured in the de novo transcriptome [25]. Use of KEGG analysis was approved with the official permission from Kanehisa laboratory.

### Small RNA sequencing analysis

Small RNA sequencing libraries were constructed using the NEXTflex Small RNA sample preparation protocol with an initial input of 70 ng of total RNA as previously described [18]. Reads were then mapped onto the human reference genome [26], using bowtie [27]. The reads for the microRNAs were counted using HTSeq [28] with the mod of ‘intersection-nonempty’ based on miRBase 20 release [29]. Given the read counts of each microRNAs, EdgeR [30] was applied to analyse the differential expression patterns. Differentially expressed microRNAs were identified with a significance of q-value <0.05 and log_2_ fold change > ±2. The raw small RNA-seq data files are accessible via GEO Series accession number GSE149983.

### miRNA-mRNA integration analysis

miRTarVis+ was used as an interactive visual analysis tool for miRNA-mRNA expression profile data [31]. miRTarVis+ uses prediction algorithms that are based on both sequence and expression profile data. miRTarVis+ supports two sequence-based prediction algorithms, TargetScan and microRNA.org, which are two of the most cited miRNA target prediction algorithms.

### Quantitative real-time RT polymerase chain reaction (qRT-PCR)

First-strand cDNA was synthesized from 0.5 μg of total RNA using ImProm-II Reverse Transcription System (Promega, Madison, WI, USA) according to manufacturer’s instructions. QuantStudio 6 Flex Real-time PCR system (Thermo Fisher Scientific, Waltham, MA, USA) was utilized for cDNA amplification with Power SYBR^®^ Green Master Mix (Invitrogen) under the following conditions: 95°C for 10 min, followed by 40 cycles of 95°C for 30 s and 60°C for 1 min. Relative mRNA levels were determined based on the ΔΔC_t_ method, and normalized against β-actin mRNA.

To validate miRNA expression changes induced by ZIKV infection, miRNAs from hUCMSCs were isolated using miRNeasy mini kit (Qiagen, Germantown, MD, USA). cDNA from miRNA was synthesized using miScript II RT kit (Qiagen) according to manufacturer’s protocol. miRNAs were detected using primers specific to miRNA (Qiagen). SNORD68 small nuclear RNA was used as internal control for normalization. qPCR was performed for detection of levels of specific miRNAs using miScript SYBR Green PCR kit (Qiagen). Quantification was carried out on QuantStudio 6 Flex Real-time PCR system.

### Transmission electron microscopy (TEM)

Mock (PBS) or ZIKV-infected hUCMSCs were pelleted and washed twice with PBS. Fixation was performed with PBS containing 2.5% glutaraldehyde and 2% paraformaldehyde for 30 min at 4°C. The pellets were rinsed twice with cold PBS, post-fixed in 1% osmium tetroxide, dehydrated in ethanol series, incubated twice with propylene oxide for 20 min and embedded in Epon mixture. Ultrathin sections of 70 nm were obtained using a Reichert-Jung Ultracut E ultramicrotome (Leica, Wetzlar), mounted on copper grids, and counterstained with uranyl acetate and lead citrate. The specimens were observed with a Hitachi H-7600 electron microscope (Hitachi, Japan) at 80 kV acceleration voltage.

### Mitochondrial metabolic flux analysis

hUCMSCs were plated on XF-96 plates (Seahorse BioSciences, Billerica, MA, USA) at a density of 8,000 cells/well. On the day of mitochondrial flux analysis, media was changed to Krebs-Henseleit buffer at pH 7.4 and incubated at 37°C in a non-CO_2_ incubator for 1 h. All media and injection reagents used in this assay were adjusted to pH 7.4. Using the XFp Extracellular Flux analyser, oxygen consumption rate (OCR) was measured as previously described [32].

### Western blotting

Cells were lysed at the specified time points using RIPA buffer (Sigma-Aldrich). Lysates were separated by sodium dodecyl sulphate polyacrylamide gel electrophoresis on 10–12% acrylamide gels. Proteins were transferred to polyvinylidene difluoride membranes and blocked with 5% (w/v) skim milk in Tris-buffered saline (0.2 M Tris, 1.36 M NaCl) supplemented with 0.1% (v/v) Tween-20 (TBS-Tw) for 1 h at 25°C. Blocking was followed by overnight incubation with primary antibodies (1:1000 dilutions, Cell signalling Technologies, Danvers, MA, USA) at 4°C. ZIKV NS1 antibody (1:1000 dilutions) was purchased from GeneTex, Irvine, CA, USA. After three washes in TBS/Tween-20, the membranes were incubated with HRP-conjugated anti-rabbit or anti-mouse IgG secondary antibodies for 1 h at 25°C. Membranes were washed with TBS/Tween-20 and incubated in Western Lumi Pico solution (ECL solution kit; DoGen, Seoul, Korea). Signals were determined using a Fusion Solo Imaging System (Vilber Lourmat, Collégien, France). Band intensities were quantified by Fusion-Capt analysis software (Vilber Lourmat).

### Transfection of plasmids, miRNA mimics and siRNAs

Tom20-GFP plasmid was kindly gifted by Dr. Woong Sun (Korea University School of Medicine) and ZIKV NS-FLAG tagged plasmids were gifted by Dr. Vaithi Arumugaswami (Addgene plasmid #79639, 79636, and 79640). For transfection of miR-142-5p mimic, cells were seeded in 12-well plates and incubated for 24 h to reach 80% confluency. Cells were transiently transfected with miR-142-5p mimic or a mimic negative control (Bioneer, Daejeon, Korea) using Lipofectamine 2000 transfection reagent (Invitrogen). After 24 h, RNA was extracted to determine transfection efficiency and knockdown by qRT-PCR. For siRNA transfection, cells were seeded in 6-well plates and cultured until 60∼80% confluency on the day of transfection. Transient transfections with control scrambled, ITGAV-, IL6ST or AXL-specific siRNAs (Bioneer) were performed with RNAiMAX transfection reagent (Invitrogen) according to manufacturer’s protocol.

### Virus entry assay

Virus entry assay was performed as previously described [33]. Cells were absorbed with ZIKV MR766 for 1 h at 4°C. After washing with cold DMEM, cells were moved to 37°C incubator for 1 h to allow for virus entry. Cells were then treated with acid glycine to inactivate non-internalized virus for 5 min and washed with cold HBSS. Without permeabilization, cells were stained with anti-pan-flavivirus envelope (E) monoclonal antibody (1:200 dilution; Abcam, Cambridge, UK), followed by anti-mouse Alexa 594 conjugated antibody (1:200 dilution; Invitrogen).

### Luciferase reporter assay

Wildtype or mutant of 3′ UTR sequences of ITGAV or IL6ST were ligated into the pRL-tk reporter plasmid (a kind gift of Dr. Hoseok Song, Korea University School of Medicine). To construct the mutant ITGAV or IL6ST 3′ UTR, the sequences that interact with the seed sequence of miR-142-5p were mutated (ITGAV: from ATACTAAAGACTT**TA**TAACTGCATGAACTT to ATACTAAAGACTT **CG** TAACTGCATGAACTT; IL6ST: from GGTAGATGAAC ACTT TA**TA**CAGTATATATCT to GGTAGATGAACACTTTA**CG**CAGTATATATCT). HEK-293 T cells were seeded in 96-well plates and co-transfected with 100 ng ITGAV or IL6ST reporter plasmids and miR-142-5p mimic or negative control using Lipofectamine 2000 transfection reagent. At 24 h post-transfection, the cells were harvested according to the manufacturer’s instructions (Promega, Madison, WI, United States), and the luciferase activities were measured using a Dual- Luciferase Reporter Assay System (Promega).

### Statistical analysis

Statistical analysis was performed using Graphpad Prism (Graphpad Software, La Jolla, CA, USA). Data are represented by means ± standard deviation (SD) and analysed using Student’s *t*-test to determine the significance of differences between the means of two groups. A two-tailed *p* value of less than 0.05 was considered significant.

## Results

### ZIKV infection in hUCMSCs

**Figure 1.**
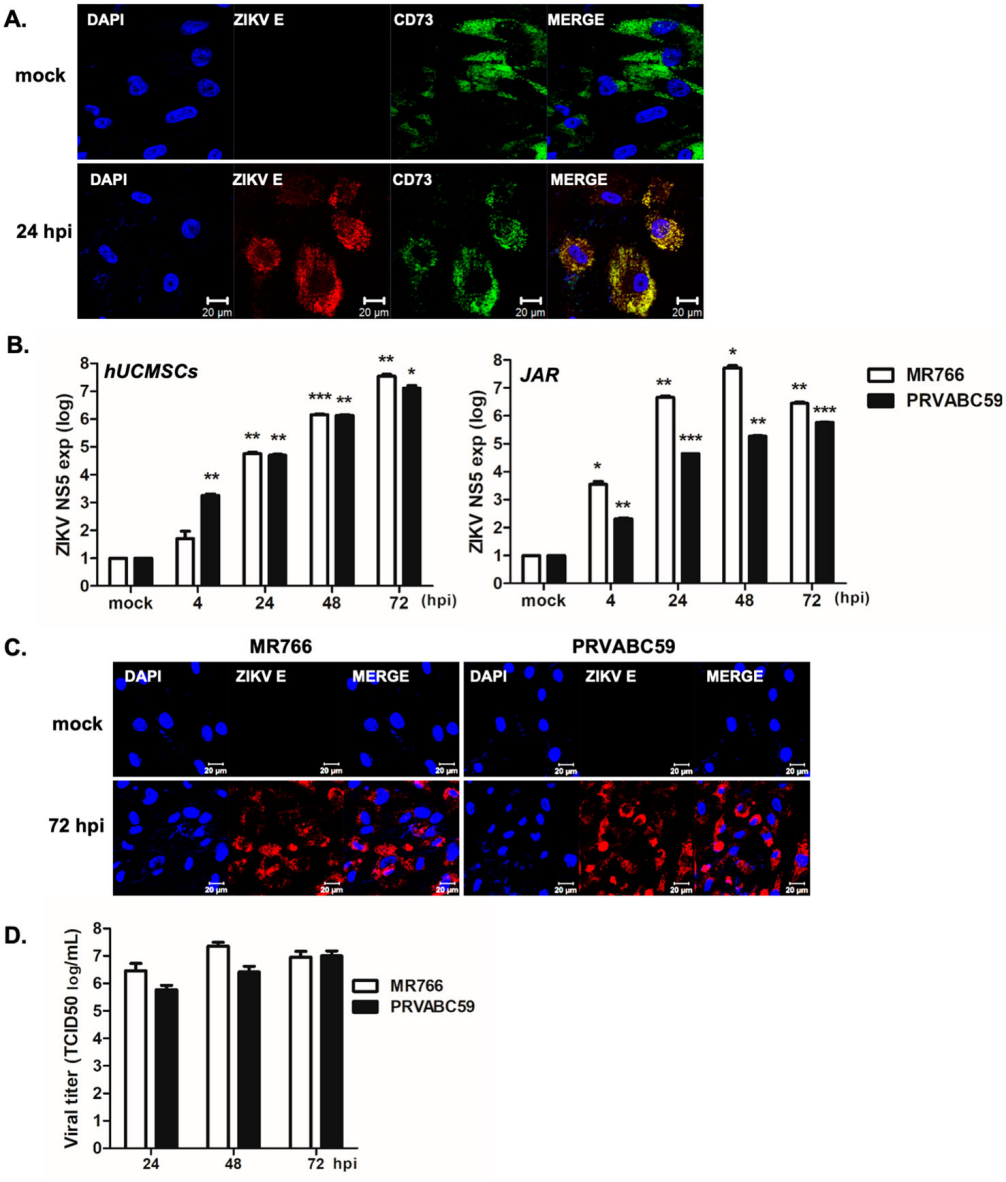
Human umbilical cord derived mesenchymal stem cells (hUCMSCs) are highly permissive to ZIKV infection (A) Confocal images of hUCMSCs infected with PRVABC59 (Asian lineage) at 24 hpi. ZIKV envelope (E) protein (Red), DAPI (blue), and CD73 (green) are shown. Scale bar = 20 μm. (B) hUCMSCs and JAR choriocarcinoma cells were infected with ZIKV at a multiplicity of infection (MOI) of 1 and RNA was collected at different time points. qRT-PCR was performed to determine the *ZIKV NS5* transcript levels. Data are representative of three independent experiments (mean ± SD). *p < 0.05; **p < 0.01; ***p < 0.001, versus mock-infected cells. (C) Cells were infected with ZIKV (MR766 or PRVABC59) at a MOI of 1 for 72 h. Cells were fixed with paraformaldehyde and permeabilized with 0.5% Triton X-100. ZIKV E protein was immunostained with anti-pan-flavivirus envelope monoclonal antibody. ZIKV E and cell nuclei are stained red and blue, respectively. The images are representative of three independent experiments. Scale bar = 20 μm. (D) Viral titers were measured by TCID50 assay. Supernatant were collected from hUCMSCs infected with ZIKV (MR766 or PRVABC59) at a MOI of 1 for various timepoints. TCID50 was calculated using the Spearman-Kärber method. Results represent means ± SD of two independent experiments.

Human umbilical cord derived MSCs (hUCMSCs) were prepared as described previously. Similar to previously reported studies [10], hUCMSCs used in our study expressed a major MSC marker, CD73 (Figure 1(A)). In addition, hUCMSCs also expressed another MSC marker, CD90, as analysed by flow cytometry. (Supplementary Figure 1(A)). Cells were infected with ZIKV (MR766 or PRVABC59) at a MOI of 1. As depicted in Figure 1(A), hUCMSCs were permissive to ZIKV infection, as shown by the detection of ZIKV protein production at 24 h post-infection (hpi). We next evaluated ZIKV replication kinetics in hUCMSCs by qRT-PCR at different time points after infection. The African lineage MR766 strain and Asian lineage PRVABC59 strain were used to infect hUCMSCs and placenta-derived choriocarcinoma cell lines (JAR) (Figure 1(B)). Expression levels of viral polymerase NS5 were measured by qRT-PCR at 4, 24, 48, and 72 hpi. ZIKV NS5 transcript levels gradually increased in both MR766 and PRVABC59-infected hUCMSCs in a time dependent manner. Consistent with previous reports of strain-specific differences in viral replication kinetics of ZIKV [34], approximately 2∼3-fold higher levels of ZIKV NS5 expression were detected in response to infection of human choriocarcinoma JAR cells with MR766 than with PRVABC59. As depicted in Figure 1(C), immunostaining of ZIKV E protein further confirmed increased rates of ZIKV infection at 72 hpi in hUCMSCs. We also measured infectivity titers of ZIKV by TCID50 assay using supernatants from hUCMSCs. Similar to qRT-PCR data, our data showed high virus infectivity titers in both MR766- and PRVABC59-infected cells in a time dependent manner (Figure 1(D)). Viral titers were moderately higher in MR766 compared with that of PRVABC59. Next, we measured and compared ZIKV-induced cytotoxicity levels in hUCMSCs and human neuronal progenitor cells (hNPCs). A slight reduction in cell viability was observed at 48 hpi in ZIKV-infected hUCMSCs in comparison to hNPCs (Supplementary Figure S1(B)). Collectively, our data indicate that hUCMSCs are highly susceptible to ZIKV infection.

### Comprehensive transcriptome analysis of ZIKV-infected hUCMSCs

**Figure 2.**
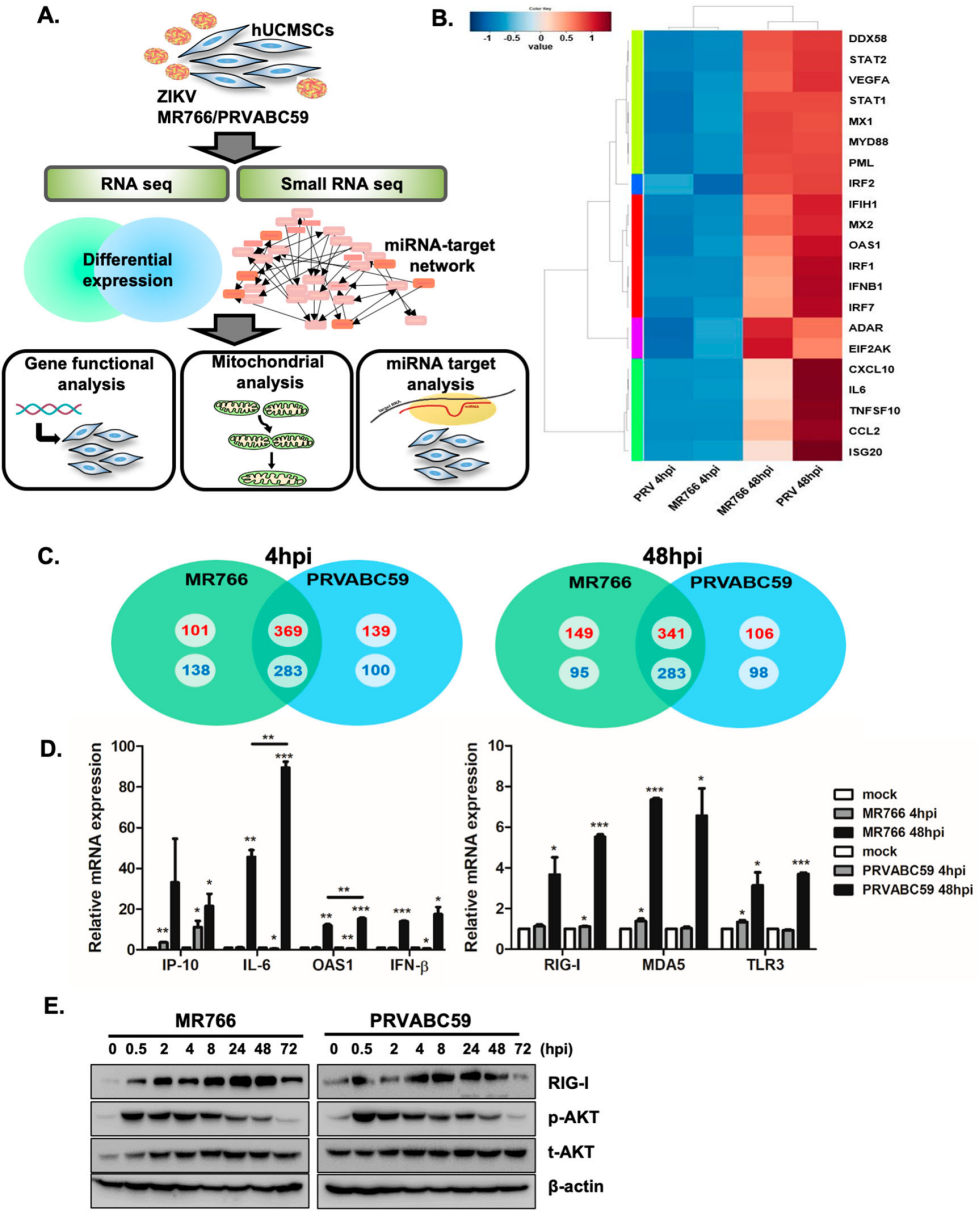
Analysis of transcriptome profiles of hUCMSCs after infection with ZIKV. (A) A schematic showing experimental design. hUCMSCs were infected with mock, MR766 and PRVABV59 (MOI of 1) for 4 and 48 hpi and RNA was analysed by RNA-seq and small RNA-seq to identify mRNA and miRNAs dysregulated by ZIKV infection. (B) Heatmaps showing the statistical over-representation of the top 20 differentially expressed genes (DEGs) involved in immune function based on the lists of transcripts that were differentially expressed (compared to the mock-infected cells). (C) Venn diagrams show numbers of more than 2 fold up or down-regulated DEGs identified from the comparison among mock and virus-infected groups. The numbers in red (upregulated) or blue (downregulated) indicate the mRNA counts in the indicated area. (D) To validate RNA-seq results, qRT-PCR was performed to measure *RIG-I, MDA5, TLR3, IP-10, IL-6, OAS* and *IFN-β* mRNA levels following ZIKV infection in hUCMSCs. Data are representative of three independent experiments (mean ± SD). *p < 0.05; **p < 0.01; ***p < 0.001, versus mock-infected cells. (E) hUCMSCs were infected with MR766 or PRVABC59 at MOI of 1. The protein levels of pAKT/AKT, RIG-I, and β-actin were measured by Western blotting.

Next we used high throughput RNA-sequencing and small RNA-sequencing to analyse differential expressions of mRNAs and miRNAs in ZIKV-infected hUCMSCs (Figure 2(A)). Each sample had relatively high sequencing coverage range. The volcano plot indicates that fold change differences correlate with significance based on *p*-values (i.e. genes with a large fold change difference had a low *p*-value in the group-wise comparison) (Supplementary Figure S2).

We compared the host transcriptional signatures in samples collected at early (4 hpi) and late (48 hpi) time points following MR766 or PRVABC59 virus infection to identify differentially expressed genes (DEGs) with a fold change of ± 2 and a q-value < 0.05. Figure 2(B) depicts a heatmap of the top 20 DEGs involved in immune function such as *CCL5, CXCL11, CXCL1, IFNB1, IL6* and *TNFSF10*. Although at 4 hpi, expressions of immune related genes were unchanged or downregulated, gene expression of anti-viral response genes were highly upregulated at 48 hpi for both MR766- and PRVABC59-infected hUCMSCs. Venn diagram identified 470 and 490 up-regulated DEGs in 4 and 48 hpi after MR766 infection respectively, whereas 508 and 447 DEGs were up-regulated in 4 and 48 hpi after PRVABC59 infection respectively. 369 and 341 upregulated DEGs were common in MR766 and PRVABC59 infections at 4 and 48 hpi, respectively (Figure 2(C)). Top 20 DEGs involved in immune function that were upregulated following ZIKV infection are shown in Supplementary Table 1, whereas top 20 down-regulated genes are listed in Supplementary Table 2. We also analysed top 20 up- or down-regulated DEGs specific to each strain of ZIKV at 48 hpi (Supplementary Table 3), but **most** of the DEGs were without any previously known functions.

To determine the gene categories broadly affected by ZIKV, Gene ontology (GO)-based bioinformatics analysis was performed. ZIKV-infected cells were differentially enriched with genes associated with the cell cycle, developmental process, and immune response (Supplementary Figure S3). DEGs were also mapped to the Kyoto Encyclopaedia of Genes and Genomes (KEGG) database, which allows mapping of known biological pathways and explicitly labelling of each proven molecular-level participant in these maps based on sequencing results. KEGG identified functional groups that were significantly enriched in response to ZIKV infection. Top 10 upregulated KEGG pathways activated in ZIKV-infected hUCMSCs include activation of “Cell cycle,” “DNA replication” and “Oxidative phosphorylation” (Supplementary Table 4). In addition, KEGG analysis of DEGs involved in the phagosome pathway showed that ZIKV infection induced expression of integrins, such as αvβ3 and αvβ5, at early timepoints (Supplementary Figure S4).

In order to confirm the RNA-seq data, qRT-PCR was used to analyse the expression levels of selected DEGs. Similar to data obtained from RNA-sequencing, qRT-PCR validated that ZIKV infection induces up-regulation of *interferon gamma induced protein 10 (IP-10), interleukin-6 (IL-6), 2'-5'-oligoadenylate synthetase 1 (OAS1)* and *interferon-β (IFN-β)* genes (Figure 2(D)). Genes that were upregulated during infection with both viruses were mainly associated with IFN signalling. Thus, we next analysed genes responsible for sensing RNA viruses in hUCMSCs. Expression levels of viral RNA sensors, such as *retinoic acid inducible gene-I (RIG-I), melanoma differentiation associated gene (MDA5), and toll-like receptor 3 (TLR3) were significantly* up regulated after ZIKV infection (Figure 2(D)). Following ZIKV infection, phospho-AKT expression was rapidly upregulated starting at 0.5 hpi and remained increased until 8 hpi. Additionally, in correlation with our RNA-seq and qRT-PCR data, RIG-I protein expression gradually increased over time, suggesting a potential role of RIG-I in sensing ZIKV RNA in hUCMSCs (Figure 2(E)). Given that RIG-I is a well-established viral RNA sensor, we investigated whether the knockdown of *RIG-I* leads to modulation of ZIKV infection and host gene expression. As shown in supplementary Figure S5, inhibition of RIG-I expression led to enhanced ZIKV *NS5* transcript levels. In addition, expression of downstream genes of RIG-I signalling was significantly reduced.

### ZIKV infection alters mitochondrial dynamics in hUCMSCs

**Figure 3.**
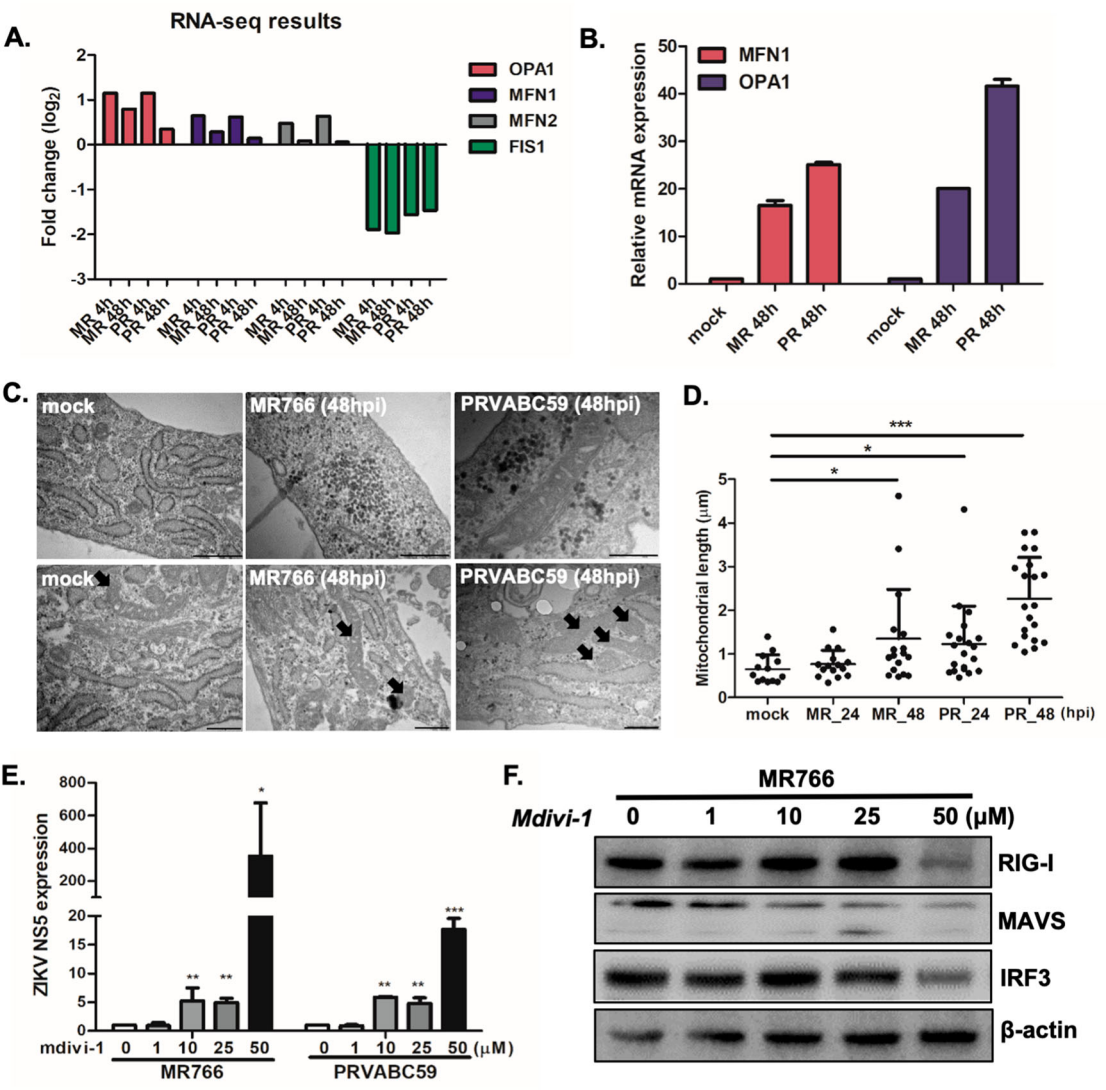
ZIKV infection results in alterations in mitochondrial morphology and function in hUCMSCs (A) Fold change levels of *OPA1, MFN1, MFN2,* and *FIS1* gene expression are shown from RNA-seq data (B) *MFN1* and *OPA1* transcript levels were measured by qRT-PCR to validate RNA-seq results shown in (A). (C) Ultra-thin section transmission electron microscopy images of mock and ZIKV-infected resin-embedded hUCMSCs (Scale bar = 500 nm). The arrows indicate mitochondria with altered morphology. ZIKV-infected cells displayed elongated mitochondria. (D) Quantitative analysis of mitochondrial lengths in mock and ZIKV-infected hUCMSCs at 24 and 48 hpi. The average mitochondrial lengths for cells within each condition were calculated using ImageJ software. Statistical analysis was done on the average mitochondrial lengths of each cell as described. *p < 0.05; **p < 0.01; ***p < 0.001, versus mock-infected cells. (E) Mdivi-1, mitochondrial fission inhibitor, was added in ZIKV-infected cells at various concentrations. qRT-PCR was performed to determine *ZIKV NS5* transcript levels. Data are representative of three independent experiments (mean ± SD). *p < 0.05; **p < 0.01; ***p  < 0.001, versus DMSO control. (F) Expression of RIG-I, MAVS and IRF3 was determined by Western blotting analysis in cell lysates of mock vs. ZIKV-infected cells.

Our transcriptome analysis demonstrate the differential expression of several genes involved in mitochondrial dynamics following ZIKV infection (Figure 3(A)). In particular, upregulation of mitochondrial fusion-related genes such as *mitofusin-1(MFN1), mitofusin-2 (MFN2),* and *OPA1 encoding dynamin-related protein,* and downregulation of mitochondrial fission-related genes like *mitochondrial fission protein 1* (*FIS1)* were observed during RNA-seq analysis. In correlation with the sequencing results, qRT-PCR data also show increased expression levels of *MFN1* and *OPA1* following ZIKV infection (Figure 3(B)). To investigate the structure of mitochondria in cells infected with ZIKV, we stained hUCMSCs with mitoTracker Green, a fluorescent dye that accumulates in active mitochondria. Confocal analysis revealed that ZIKV-infected cells exhibited larger and fused mitochondria at 48 hpi, whereas mock-infected control cells showed both fused and fragmented mitochondria (Supplementary Figure S6).

Transmission electron microscopy (TEM) also indicated that ZIKV-infected cells mostly showed fused and elongated mitochondria at 48 hpi (Figure 3(C)), similar to DENV infection [35, 36]. Quantification of TEM analysis demonstrated that ZIKV infection induces an increase in mitochondrial length per cell at 48 hpi (Figure 3(D)). To determine whether the alterations in mitochondrial morphology during ZIKV infection affect mitochondrial function, we measured the oxygen consumption rate (OCR), an indicator of cellular respiration, in mock- and ZIKV-infected hUCMSCs. Compared to mock-infected cells, ZIKV-infected cells displayed increased basal levels of OCR, which suggests that ZIKV induces increased respiration in hUCMSCs (Supplementary Figure S7). Oxygen consumption rate (OCR) in mock-infected and ZIKV-infected hUCMSCs was measured in real-time under basal conditions by XF Seahorse analyser. OCR was performed every 7 min following ZIKV infection in real time. We measured basal OCR level within 2 h after ZIKV infection. There was no cytotoxic effect by ZIKV infection at 2 h, causing no change in cell density. To address the relevance of the alterations in mitochondrial dynamics in ZIKV infection, we treated hUCMSCs with various concentrations of Mdivi-1, a mitochondrial fission inhibitor. Mdivi-1 treatment led to a dose-dependent increase in ZIKV NS5 transcript levels, suggesting that mitochondrial fusion may facilitate viral replication (Figure 3(E)). To characterize the detailed mechanisms by which mitochondrial fusion may alter viral replication, expression levels of RIG-I, mitochondrial antiviral signalling protein (MAVS), and interferon regulatory factor 3 (IRF3) in mdivi-1-treated cells were measured by Western blot analysis. Figure 3(F) shows that high dose of mdivi-1 leads to the suppression of RIG-I, MAVS and IRF3 expression levels, suggesting that impairment of mitochondrial fission in ZIKV-infected hUCMSCs can be a part of the immune evasion strategies utilized by ZIKV in order to facilitate viral pathogenesis (Figure 3(F)).

### Distinct cellular miRNA expression profiles in response to ZIKV infection

**Figure 4.**
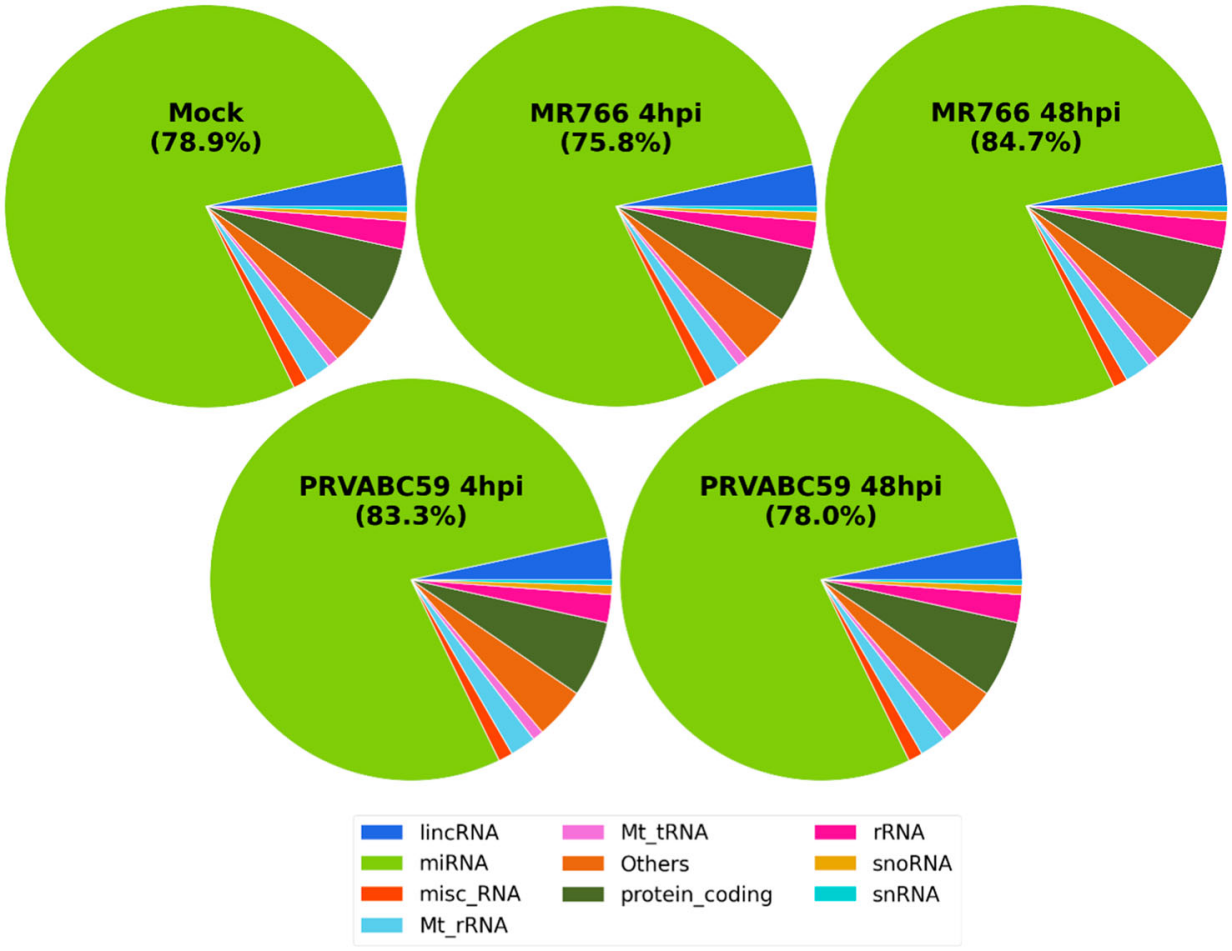
Comprehensive analysis of small RNAs during ZIKV infection in hUCMSCs. Pie chart of mappable small RNAs obtained by small RNA next generation sequencing in ZIKV-infected hUCMSCs. Small RNA-seq reads are distributed across categories of annotated small RNAs pre-miRNAs, small non-coding RNAs (snoRNAs), long non-coding RNAs (lncRNAs), transfer RNAs (tRNAs), ribosomal RNAs (rRNAs) in mock-treated or ZIKV MR766- or PRVABC59-infected cells at 4 and 48 hpi.

MicroRNAs (miRNAs) are non-coding RNAs known to regulate various cellular process [37]. The Illumina genome analyser platform was used for high throughput sequencing of short cDNA libraries corresponding to 8-to 35-nucleotide RNAs. Only miRNAs that experienced at least q-value <0.05 and log_2_ fold change > ±2 were considered as significant. Pie charts were drawn to depict the frequency of different classes of RNA species present in the small RNA datasets. As expected, the majority of small RNA-seq reads were mapped to human encoded miRNAs, as well as long non-coding RNAs and small non-coding RNAs (Figure 4). We then compared the expression patterns of miRNAs in MR766 and PRVABC59 infected hUCMSCs at 4 and 48 hpi. More than 13 million filtered high quality reads, representing more than 500 known miRNAs, were obtained for both MR766 and PRVABC59-infected cells (Supplementary Table 5). Top 10 differentially up-regulated and down-regulated miRNAs in cells infected with MR766 and PRVABC59 are listed in Tables 1 and 2. At 48 hpi, 13 and 18 miRNAs were up- or downregulated by more than 2 fold in MR766-infected cells and 7 and 21 miRNAs were up- or downregulated by more than 2 folds in PRVABC59-infected cells, respectively.

**Table 1.** Top 10 up-regulated miRNAs in ZIKV-infected hUCMSCs.

	MR766, 4 hpi			MR766, 48 hpi			PRVABC59, 4 hpi			PRVABC59, 48 hpi		
	miR	FC*	*p* value	miR	FC*	*p* value	miR	FC*	*p* value	miR	FC*	*p* value
1	hsa-miR-1287-5p	6.07	0.0102	hsa-miR-323a-5p	6.35	0.0128	hsa-miR-3913-5p	6.51	0.0071	hsa-miR-9-3p	6.56	0.0047
2	hsa-miR-3913-3p	5.98	0.0119	hsa-miR-1261	5.31	0.0289	hsa-miR-3180-5p	6.22	0.0094	hsa-miR-1287-5p	5.27	0.0169
3	hsa-miR-323a-5p	5.95	0.0113	hsa-miR-548n	5.10	0.0339	hsa-miR-548n	5.61	0.0166	hsa-miR-7704	5.24	0.01752
4	hsa-miR-3913-5p	5.41	0.0192	hsa-miR-1287-5p	5.07	0.0348	hsa-miR-4773	5.58	0.0171	hsa-miR-876-3p	5.21	0.01816
5	hsa-miR-1908-3p	5.02	0.0276	hsa-miR-9-3p	5.07	0.0348	hsa-miR-548f-3p	5.58	0.0171	hsa-miR-365a-5p	5.10	0.02033
6	hsa-miR-381-5p	5.02	0.0276	hsa-miR-1185-5p	5.0435	0.0359	hsa-miR-4683	5.34	0.0212	hsa-miR-4683	4.80	0.02754
7	hsa-miR-1910-3p	4.98	0.0288	hsa-miR-153-3p	5.04	0.0359	hsa-miR-1913	5.30	0.0219	hsa-miR-4511	4.71	0.02892
8	hsa-miR-3617-5p	4.98	0.0288	hsa-miR-219a-2-3p	4.83	0.0420	hsa-miR-1287-5p	5.22	0.0235	hsa-miR-548az-5p	4.66	0.0304
9	hsa-miR-7976	4.67	0.0383	hsa-miR-655-5p	4.83	0.0420	hsa-miR-4781-3p	5.22	0.0235	hsa-miR-323a-5p	4.60	0.0320
10	hsa-miR-1289	4.61	0.0404	hsa-miR-6507-5p	4.75	0.0435	hsa-miR-138-1-3p	4.91	0.0310	hsa-miR-589-3p	4.60	0.0320

FC*: fold change.

**Table 2.** Top 10 down-regulated miRNAs in ZIKV-infected hUCMSCs.

	MR766, 4 hpi			MR766, 48 hpi			PRVABC59, 4 hpi			PRVABC59, 48 hpi		
	miR	FC	*p* value	miR	FC	*p* value	miR	FC	*p* value	miR	FC	*p* value
1	hsa-miR-6511a-5p	−6.25	0.0084	hsa-miR-6511a-5p	−6.70	0.0134	hsa-miR-6511b-5p	−6.91	0.0046	hsa-miR-6511a-5p	−6.43	0.0062
2	hsa-miR-1267	−5.67	0.0144	hsa-miR-548o-3p	−6.27	0.0189	hsa-miR-6511a-5p	−6.22	0.0089	hsa-miR-3179	−5.81	0.0114
3	hsa-miR-3177-3p	−5.39	0.0186	hsa-miR-1304-5p	−5.54	0.0339	hsa-miR-4435	−5.64	0.0153	hsa-miR-550a-3p	−5.73	0.0127
4	hsa-miR-1304-5p	−5.10	0.0245	hsa-miR-4461	−5.22	0.0435	hsa-miR-3179	−5.68	0.0157	hsa-miR-1197	−5.60	0.0143
5	hsa-miR-548ag	−4.78	0.0334	hsa-miR-548ag	−5.22	0.0435	hsa-miR-616-3p	−5.61	0.0157	hsa-miR-5701	−5.31	0.0188
6	hsa-miR-3680-5p	−4.67	0.0364	hsa-miR-3680-5p	−5.11	0.0468	hsa-miR-6511a-3p	−5.26	0.0219	hsa-miR-5096	−4.90	0.0289
7	hsa-miR-3928-3p	−4.67	0.0364	hsa-miR-142-5p	−5.05	0.0486	hsa-miR-1304-5p	−5.06	0.0263	hsa-miR-26a-1-3p	−4.84	0.0304
8	hsa-miR-3199	−4.55	0.0404				hsa-miR-30c-1-3p	−5.06	0.02630	hsa-miR-3680-5p	−4.84	0.0304
9	hsa-miR-548a-3p	−4.55	0.0404				hsa-miR-301a-3p	−4.79	0.0339	hsa-miR-3199	−4.73	0.0338
10	hsa-miR-579-3p	−4.55	0.0404				hsa-miR-142-5p	−4.69	0.0374	hsa-miR-514b-5p	−4.73	0.0338

**Figure 5.**
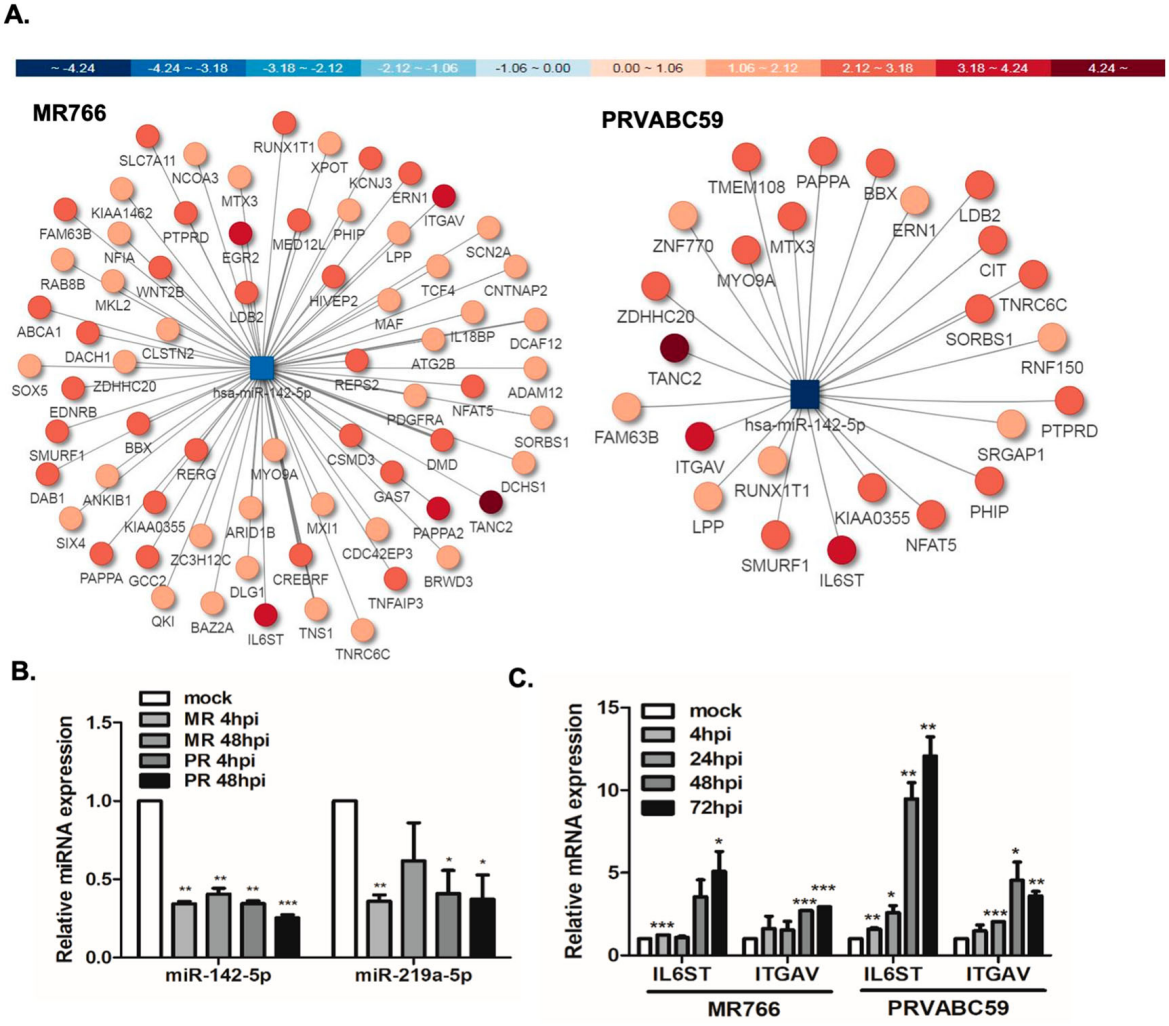
miRNA-mRNA associated network analysis present targets of miR-142-5p. (A) miRNA–mRNA associated network for ZIKV-infected hUCMSCs are shown. miRNA (square) and their predicted target mRNAs (circles) are connected by lines. The colour of each miRNA and mRNA is annotated by Pi score. Hubs were devised on the basis of miRNAs that shared multiple common mRNA targets. Downregulated miR-142-5p regulates many putative target genes, which were found to be upregulated by ZIKV infection. (B) Expressions of miR-142-5p and miR-219a-5p in hUCMSCs were verified by qRT-PCR using specific primers for the mature form of miRNAs. Similar expression levels of miRNAs were observed in the small RNA-seq results. *p < 0.05; **p < 0.01; ***p < 0.001, versus mock-infected cells. (C) mRNA expression of *IL6ST* and *ITGAV* was measured in mock and ZIKV-infected hUCMSCs using qRT-PCR. *p < 0.05, **p < 0.01, and ***p < 0.001, compared with mock control.

To further elucidate the function of differentially expressed miRNAs in ZIKV infection, biological analysis of mRNA targets of the significantly regulated miRNAs was carried out using TargetScan and microRNA.org. Among the top ten most differentially expressed miRNAs, miR142-5p was selected for further investigation following a comprehensive analysis of the predicted targets. The map of miRNA-mRNA network analysis show downregulated miRNAs (square) targeting the upregulated putative mRNA targets (circles) based on miRTarVis+ analysis. Among many predicted targets of miR-142-5p, *IL6ST* and *ITGAV* expression and function were further analysed (Figure 5(A)). *IL6ST* is an IL-6 signal transducer, which plays a critical role in regulating myocyte apoptosis, whereas *ITGAV* is an integrin subunit alpha V, which functions in cell surface adhesion and signalling. To validate the small RNA-seq data of differentially regulated miRNAs, a subset of miRNAs that were commonly downregulated by both MR766 and PRVABC59 strains was selected for further studies using qRT-PCR. In accordance with the small RNA-seq profiles, both miR-142-5p and miR-219-5p decreased significantly upon ZIKV infection (Figure 5(B)). Consistent with the data from miRNA-mRNA network analysis, qRT-PCR data showed that the expression of miR-142-5p negatively correlated with the mRNA levels of *IL6ST* and *ITGAV* in both ZIKV-infected hUCMSCs (Figure 5(C)).

### miR-142-5p regulates ZIKV replication through the target genes IL6ST and ITGAV

**Figure 6.**
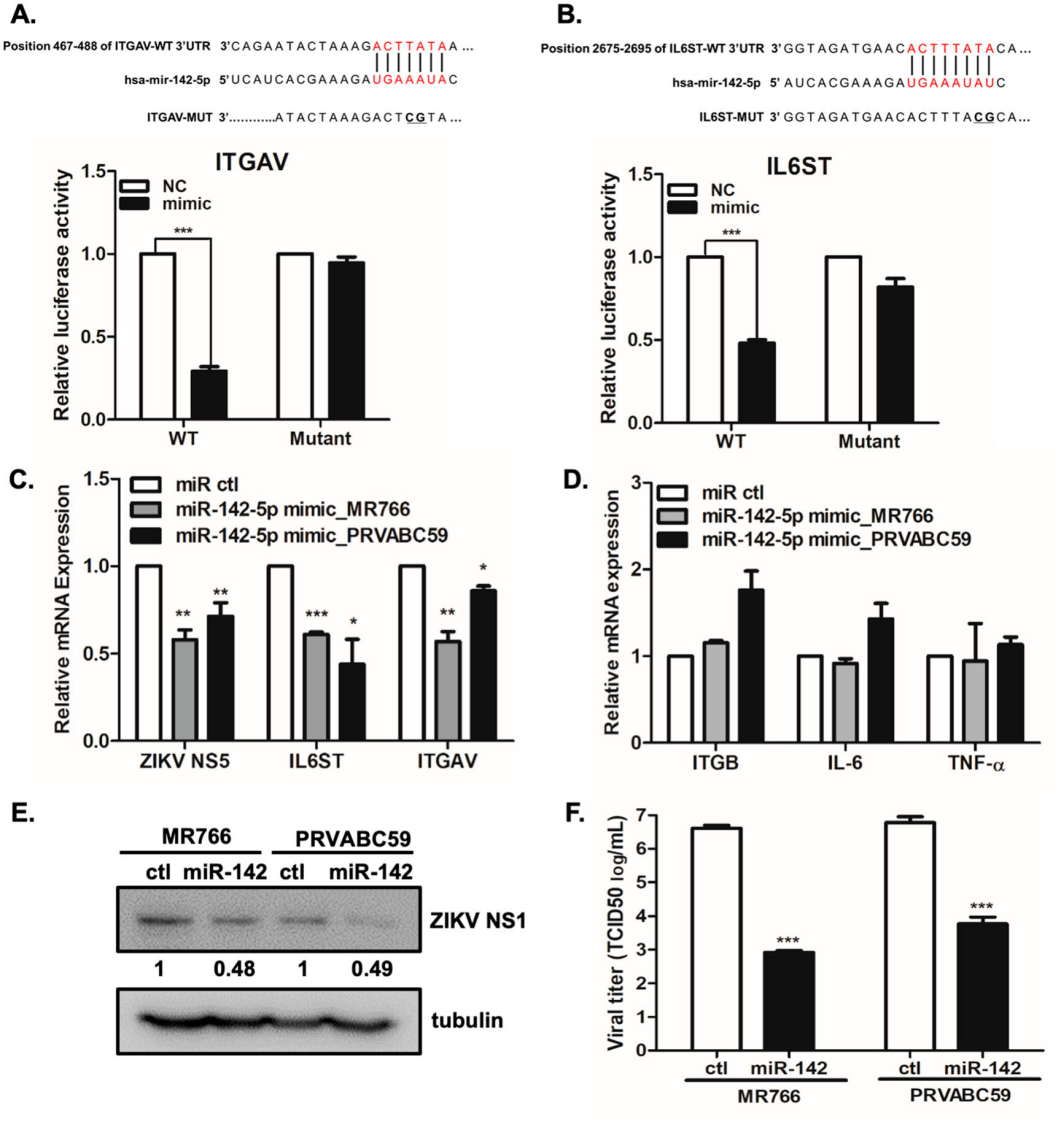
IL6ST and ITGAV are targets of miR-142-5p and overexpression of miR-142-5p results in the reduction of ZIKV replication. (A, B) Schematic presentation of the predicted miR-142-5p binding sites within the ITGAV (A) and IL6ST (B) 3’UTR and a mutated type of ITGAV and IL6ST, as predicted by the Target Scan database. The mutated nucleotides are marked in bold. Luciferase reporter assay performed using HEK293 T cells transfected with plasmids into which the luciferase reporter gene was fused to fragment of wild type or mutant 3’UTRs of ITGAV and IL6ST. (C, D) Cells were transfected with miR-142-5p mimic or mimic negative control as indicated. 24 h later, cells were infected with ZIKV (MR766 or PRVABC59) at MOI of 1 for 24 h. (C) *IL6ST*, *ITGAV,* and *ZIKV NS5* transcript levels were determined by qRT-PCR. (D) mRNA expression of *ITGB1, IL-6,* and *TNF-α* was measured by qRT-PCR. *p < 0.05, **p < 0.01, and ***p < 0.001, compared with control mimic. (E) Expression of ZIKV NS1 was examined by Western blot analysis. Quantitative densitometric analysis of Western blot analysis is presented, with normalized densitometric units plotted against treatment (shown as numbers). (F) Measurement by TCID50 assay of infectious ZIKV release into supernatant at 48 hpi is shown (MOI 1). Results represent means ± SD of two independent experiments.

To confirm if IL6ST and ITGAV are directly targeted and regulated by miR-142-5p, luciferase reporter genes with IL6ST or ITGAV of 3’ UTR and the mutant counterparts at the miR-142-5p binding regions were co-transfected with miR-142-5p mimic. The putative miR-142-5p binding sites in IL6ST and ITGAV 3’UTR and mutant IL6ST and ITGAV 3’UTR with modified binding sequences are shown in Figure 6(A and B). Overexpression of miR-142-5p significantly inhibited the luciferase activity of IL6ST and ITGAV with the wild type 3’ UTR, but not with the mutant 3’UTR, demonstrating that miR-142-5p directly targets IL6ST and ITGAV (Figure 6(A and B)).

To examine the potential function of miR142-5p during ZIKV infection, we further investigated the effect of miR-142-5p mimic on ZIKV replication in A549 cells. Overexpression of miR-142-5p in ZIKV- infected A549 cells significantly attenuated *ZIKV NS5* gene expression, while it markedly reduced *IL6ST* and *ITGAV* expressions at the mRNA level (Figure 6(C)), while no effects were observed on off-target genes such as *integrin β1 (ITGB1), IL6,* and *TNF-α* (Figure 6(D)). To validate qRT-PCR data, we also examined the effect of miR-142-5p mimic on ZIKV NS1 protein expression by Western blot analysis. We performed quantitative densitometric analysis of Western blot analysis to quantify the protein expression levels. As shown in Figure 6(E), there was more than 50% decrease in ZIKV NS1 expression levels following miR-142-5p mimic treatment compared with that of control mimic treated cells. Therefore, the Western blot data is positively correlated with qRT-PCR data, suggesting significant change in viral replication by miR-142-5p mimic treatment. The release of live viral particles was also quantified by TCID50 assay, and virus titers of miR-142-5p mimic-transfected cells were suppressed in comparison to those of control transfection (Figure 6(F)).

**Figure 7.**
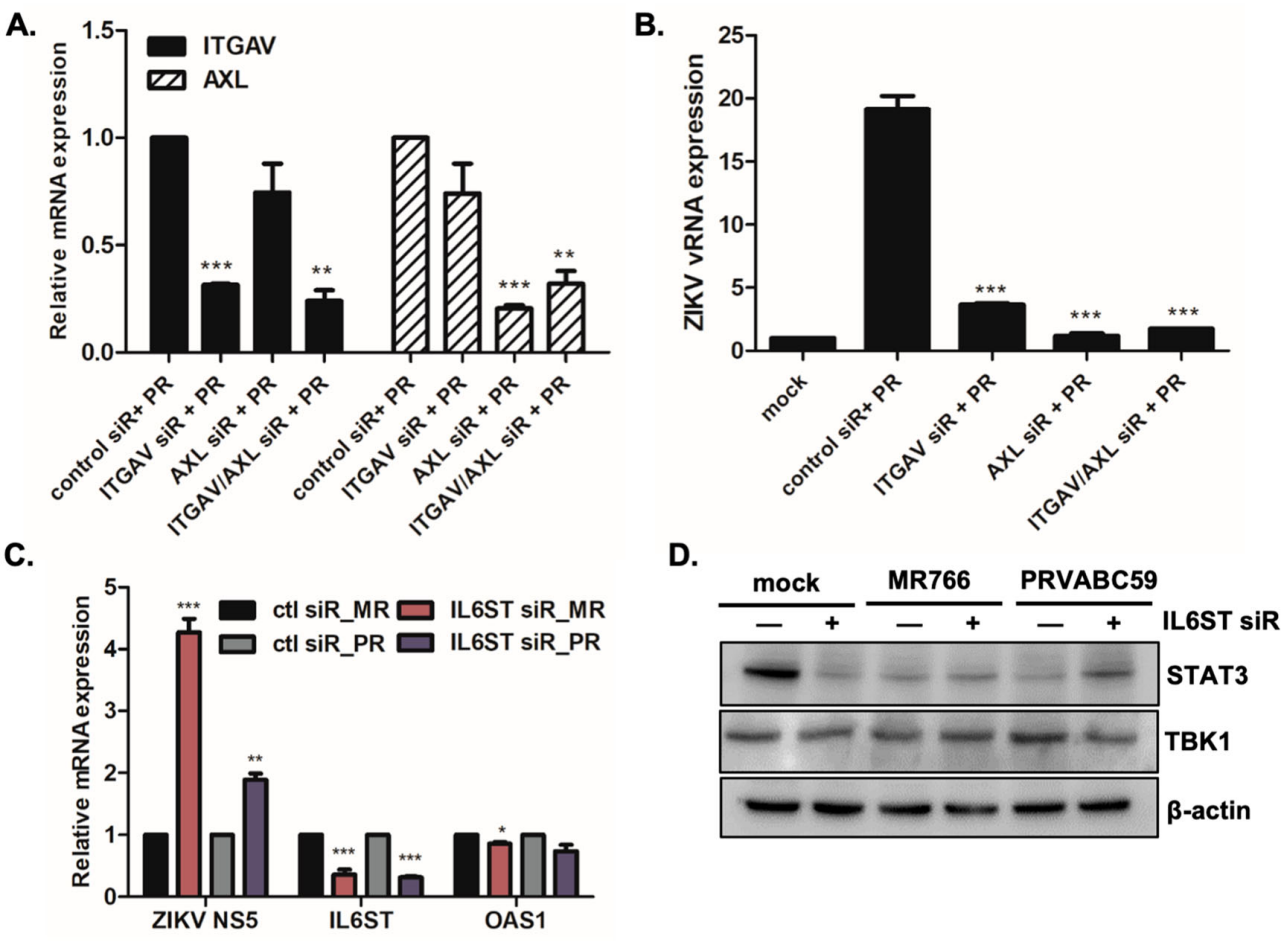
The effect of miR-142-5p targets, ITGAV and IL6ST, on ZIKV infection. (A, B) A549 cells were transfected with control or *ITGAV or AXL*-specific siRNA followed by ZIKV infection (PRVABC59) at MOI of 1 for 24 h. The knockdown efficiency of *ITGAV or AXL*-specific siRNA treatment was measured by qRT-PCR. (B) Expression levels of *ZIKV vRNA* were measured by qRT-PCR. The results represent mean ± SD. *p < 0.05, **p < 0.01, and ***p < 0.001, compared with control siRNA-treated cells. (C) A549 cells were transfected with control or *IL6ST*-specific siRNA followed by ZIKV infection (MR766 or PRVABC59) at MOI of 1 for 24 h. Expression levels of *ZIKV NS5, IL6ST* and *OAS1* were measured by qRT-PCR. Data are representative of three independent experiments (mean ± SD). *p < 0.05, **p < 0.01, and ***p < 0.001, compared with control siRNA-transfected cells. (D) Expression of STAT3, TBK1, and β-actin was determined by Western blotting analysis in cell lysates of mock vs ZIKV-infected cells.

For further evaluation of the effects of IL6ST and ITGAV on viral replication efficiency, IL6ST or ITGAV siRNA was transfected into A549 cells. Given that AXL is a well-known entry receptor for ZIKV, AXL siRNA was used as a positive control. First, qRT-PCR results showed downregulation of both IL6ST and ITGAV expression following specific siRNA treatment (Figure 7(A and C)). Blocking ITGAV by siRNA transfection resulted in significantly suppressed gene expression of ZIKV RNA (Figure 7(B)). Furthermore, we performed viral binding assay to determine whether miR-142 enhances viral binding/entry through ITGAV. Supplementary Figure S9 indicates that ITGAV-specific siRNA treatment led to diminished ZIKV E binding to cells. This finding suggests a potential role of ITGAV in ZIKV entry. We also investigated the effect of IL6ST on ZIKV replication. Interestingly, knockdown of IL6ST significantly upregulated ZIKV replication, highlighting the possible anti-viral function of IL6ST (Figure 7(C)). The effect of IL6ST siRNA treatment downstream of IL6ST signalling was also investigated by measuring the level of STAT3 expression using western blot. Reduced expression of STAT3 was observed following IL6ST siRNA transfection, suggesting that IL6ST also affects JAK/STAT signalling pathways (Figure 7(D)).

## Discussion

In light of the rapid spread of ZIKV epidemic since 2015, a global collaborative effort has been made to understand the mechanisms of ZIKV maternal-fetal transmission. However, to this date, earliest events of ZIKV transmission in the maternal uterine environment have yet to be fully determined. In this study, we identified significantly modulated mRNAs and miRNAs during the course of ZIKV infection in the hUCMSCs using next-generation sequencing. Our data revealed a similar pattern in the regulation of common mRNA and miRNAs between MR766 and PRVABVC59-infected cells. Using bioinformatics analysis coupled with gene expression profiles, we identified various host pathways involved in antiviral immunity and mitochondrial dynamics potentially involved in ZIKV pathogenesis. Further, our data suggest that miR-142-5p along with its target, IL6ST and ITGAV, plays an important role in the regulation of ZIKV replication.

ZIKV can be mainly classified into the African and Asian lineages, the latter of which has been responsible for the recent epidemics. Several groups have noted phenotypic differences between Asian and African lineage ZIKV strains depending on the cell type. In particular, infection with African lineage strains of human neuronal stem cells resulted in a higher rate of infection and virus production, as well as elevated degree of cell death and antiviral response [38–42]. Similar findings were observed in African-lineage ZIKV-infected trophoblasts [43]. Consistent with previous studies, our results also suggest that MR766-infected cells showed relatively higher rates of infection than PRVABC59-infected cells (Figure 1). In terms of the host transcriptome, top 20 upregulated DEGs were enriched in functions related to anti-viral immunity. The similarities, in terms of both quantity and magnitude, between the expression profiles of these top 20 upregulated DEGs are likely due to the similar rates of viral replication observed within hUCMSCs. Interestingly, both RNA-sequencing and qRT-PCR assay validated that *IP-10, IL-6, OAS1*, and *IFN-β* gene expression levels were elevated to a greater extent (1.5∼2 fold) in PRVABC59-infected cells than in MR766-infected cells at 48 hpi (Figure 2(D)). Thus, it is highly possible that enhanced anti-viral response following PRVABC59 infection contributed to the low viral replication efficiency in MR766-infected cells.

Our transcriptome analysis highlighted an upregulation of several genes involved in mitochondrial dynamics such as *MFN1, MFN2*, and *OPA1* (Figure 3(A)). Mitochondrial dynamics can be a major determinant of a viral infection outcome as they influence cellular homeostasis, metabolism and innate immune signalling [44]. Other flaviviruses such as DENV and Japanese encephalitis virus have been shown to trigger structural and functional alterations in mitochondria. In particular, DENV infection induces mitochondrial elongation by blocking dynamin-related protein 1 triggered mitochondrial fission [35, 36]. Specifically, NS4b of DENV induces mitochondrial elongation by blocking Drp1 activation [45]. Similarly, NS2b3 of DENV partially cleaved mitofusin 1 (Mfn1) and 2 (Mfn2), leading to the reduction of IFN activation [36]. In correlation with this, we also observed that NS4b of ZIKV co-localizes with a mitochondrial marker (Supplementary Figure S8) and mitochondria of ZIKV-infected cells had an elongated morphology compared with mitochondria of mock-infected cells (Figure 3). Moreover, real-time measurement of mitochondrial respiration profiling exhibited an increase in oxygen consumption rate in ZIKV-infected cells compared with mock-infected controls. Given that mdivi-1 (mitochondrial fission inhibitor) treatment enhanced viral gene expression and reduced RIG-I-mediated activation of IFN signalling pathways, mitochondrial elongation driven by ZIKV infection may dampen the early IFN response in favour of viral replication. Further studies on which ZIKV proteins alter virus-modulated morphodynamics of the mitochondria are necessary to understand the strategies utilized by ZIKV to subvert the innate immune response.

Few studies have shown that ZIKV infection induces changes in the expression levels of miRNAs, including miR-30e-3p, miR-30e-5p, and miR-17-5p in astrocytes [46], and miR-124-3p in human neural stem cells [47]. Here, our study identified miR-142-5p as a potential contributing factor that controls ZIKV pathogenesis. Transfection with hsa-miR-142-5p mimic in A549 cells effectively reduced ZIKV replication, as evident from the reduced ZIKV NS5 transcript and NS1 protein levels in the presence of the mimic (Figure 6). In support of our findings, a recent report by Berrien-Elliott et al. suggested miR-142 as an important anti-viral defence player that maintains homeostasis and function of type I innate lymphoid cells [48]. Given that miR-142 is enriched in hematopoietic tissue [49], this miRNA may be involved in the lineage differentiation of hematopoietic cells. Interestingly, miR-142 has gained considerable attention for its essential role in regulating the immune response. For example, Wong et al. has reported that a fever can lead to the induction of miR-142-5p, which targets many immune-related genes, such as *IL6ST, TNF, toll-like receptor 2,* and *prostaglandin E receptor 2* [50]. Considering that miR-142-5p may be a critical modulator of viral pathogenesis, further studies are necessary to understand the molecular mechanisms through which miR-142 mediate cellular response to ZIKV.

We also analysed the potential role of putative miR-142-5p target genes, IL6ST and ITGAV, during ZIKV infection. IL6ST (gp130) is a signal transduction molecule that mediates downstream signalling of IL-6 family of cytokines. Harker et al. emphasizes the importance of gp130 signalling in T cells, in terms of efficient T cell response against viral infection, indicating a potential antiviral role for IL6ST [51]. In accordance, our result also suggest that IL6ST may activate JAK/STAT pathway-mediated antiviral immunity to suppress ZIKV replication. Another target of miR-142 is integrin subunit alpha V (ITGAV). In supplementary Figure 4, KEGG analysis of upregulated DEGs revealed an enrichment of the phagosome pathway and upregulation of integrin *αvβ3* and *αvβ5* during ZIKV infection at 4 hpi. As highly conserved molecules expressed by almost every cell type, integrins serve as entry receptors of several viruses, including Adenovirus [52], Foot-and-mouth disease virus [53], hantavirus [54], human coxsackievirus A9 [55], human echovirus 9 [56], and WNV [57]. A very recent study by Wang et al. identified integrin avb5 as an entry receptor for ZIKV to establish an infection in neural stem cells [58]. Similarly, our data from ZIKV entry assay confirm that ITGAV is essential for the initiation of ZIKV internalization. It will be interesting to examine whether other integrins play a role in viral binding and entry, and if integrins work together with AXL receptor during ZIKV entry.

Overall, this study provides a comprehensive view of the changes in host mRNA and miRNAs induced by ZIKV infection and highlights the importance of miRNAs in the discovery and characterization of cellular factors involved in the modulation of virus replication.

## Supplementary Material

Clean_copy_of_supplementary_files.docx
