# Supplementary material for: mRNA and miRNA profiling of Zika virus-infected human umbilical cord mesenchymal stem cells identifies miR-142-5p as an antiviral factor
Source: Emerg Microbes Infect. 2020 Sep 22;9(1):2061–75. doi: 10.1080/22221751.2020.1821581 (PMC7534337; doi:10.1080/22221751.2020.1821581)
Supplement: Clean_copy_of_supplementary_files.docx [file TEMI_A_1821581_SM9118.docx]

**Supplementary figure S1. Cellular characteristics of ZIKV-infected hUCMSCs**

A) Flow cytometric analysis of cell surface antigen expression (CD90) on hUCMSCs. Cells were extensively washed in PBS and immunostained using anti-CD90 antibody (fluorescein isothiocyanate, 1:50 dilution, BD Pharmingen), diluted in FACS buffer (PBS, 0.2% BSA). Analyses were performed on a BD LSR-FORTESSA cytometer. Data were further analysed using FlowJo software 10.1.

B) hUCMSCs or human neuronal progenitor cells (hNPCs) were infected with ZIKV (MR766 or PRVABC59) for 48hpi. Cell viability was determined using Cell titer Glo assay at different MOIs. Data are representative of three independent experiments, each performed in triplicate (error bars represent SD). Statistical analysis: **p < 0.05; **p < 0.01; ***p < 0.001* versus mock-infected control cells.

**
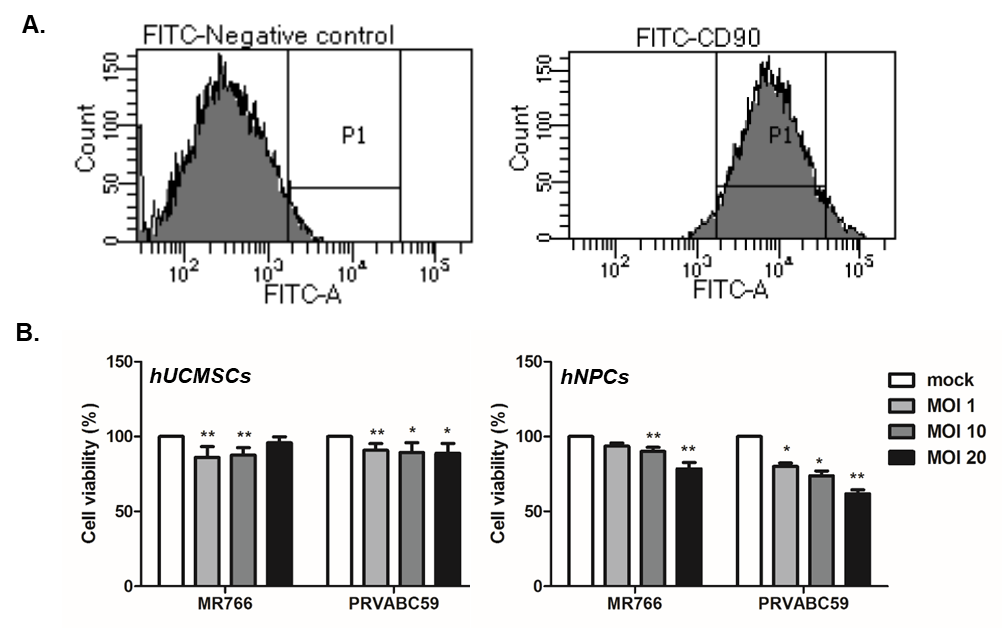
**

**Supplementary figure S2. Volcano plots for MR766 or PRVABC59-infected hUCMSCs.** The x-axis represents the log_2_ values of the fold change observed for each mRNA transcript, and the y-axis represents the -log_10_ values of the *p*-values of the significance tests between replicates for each transcript. Genes that were not differentially expressed are plotted in black.


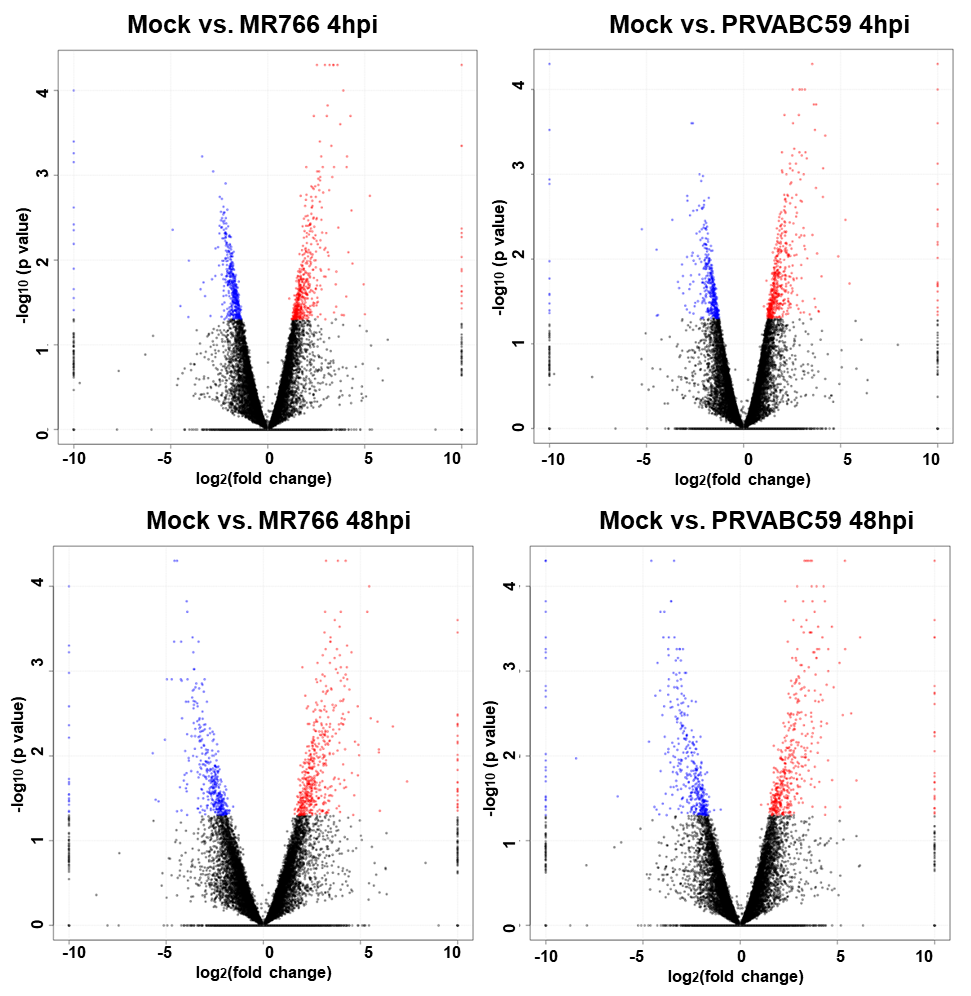


**Supplementary figure S3. Gene Ontology (GO)-based functional enrichment analysis of DEGs in ZIKV-infected hUCMSCs**

Gene Ontology (GO)-based functional enrichment analysis reveal biological processes enriched and potentially regulated at 4 hpi (A) 24 hpi (B) 48 hpi (C) and 72 hpi (D).


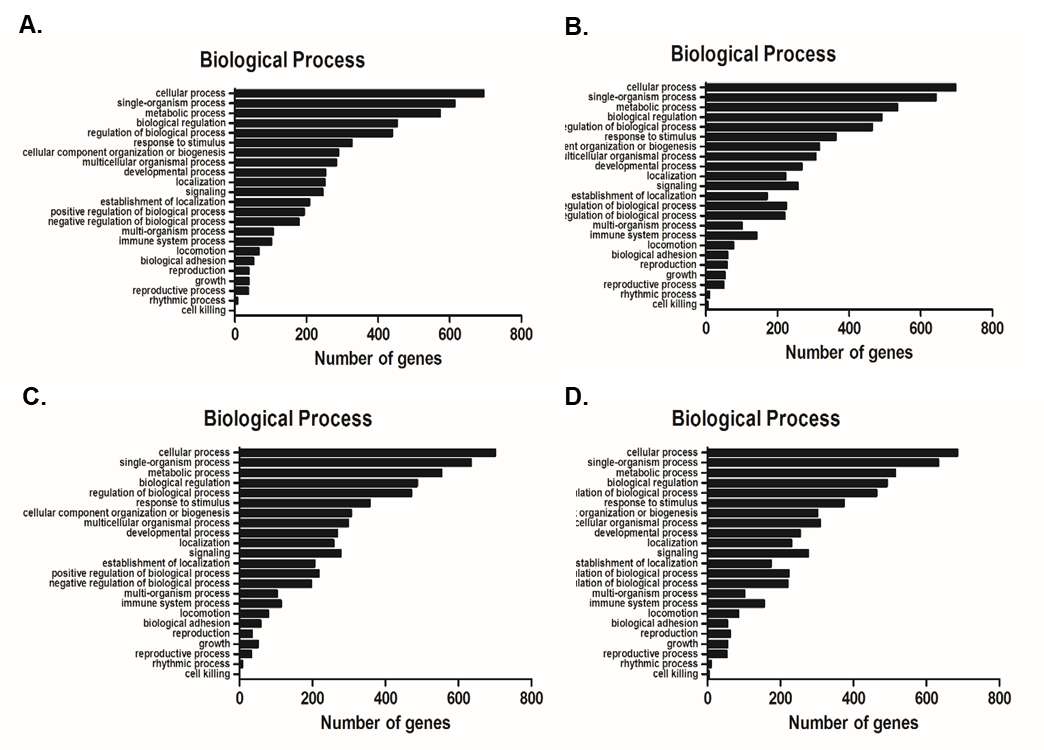


**Supplementary figure S4. Phagosome formation pathway by KEGG analysis**

KEGG analysis shows the list of DEGs involved in phagosome formation pathways. The red, green and white colours indicate significantly increased, significantly decreased, and unchanged gene expression between mock vs. MR766-infected cells (A), mock vs. PRVABC59-infected cells (B), respectively. DEGs were labelled into the map of phagosome formation pathway obtained from KEGG database with official permission and guidance [1-3].
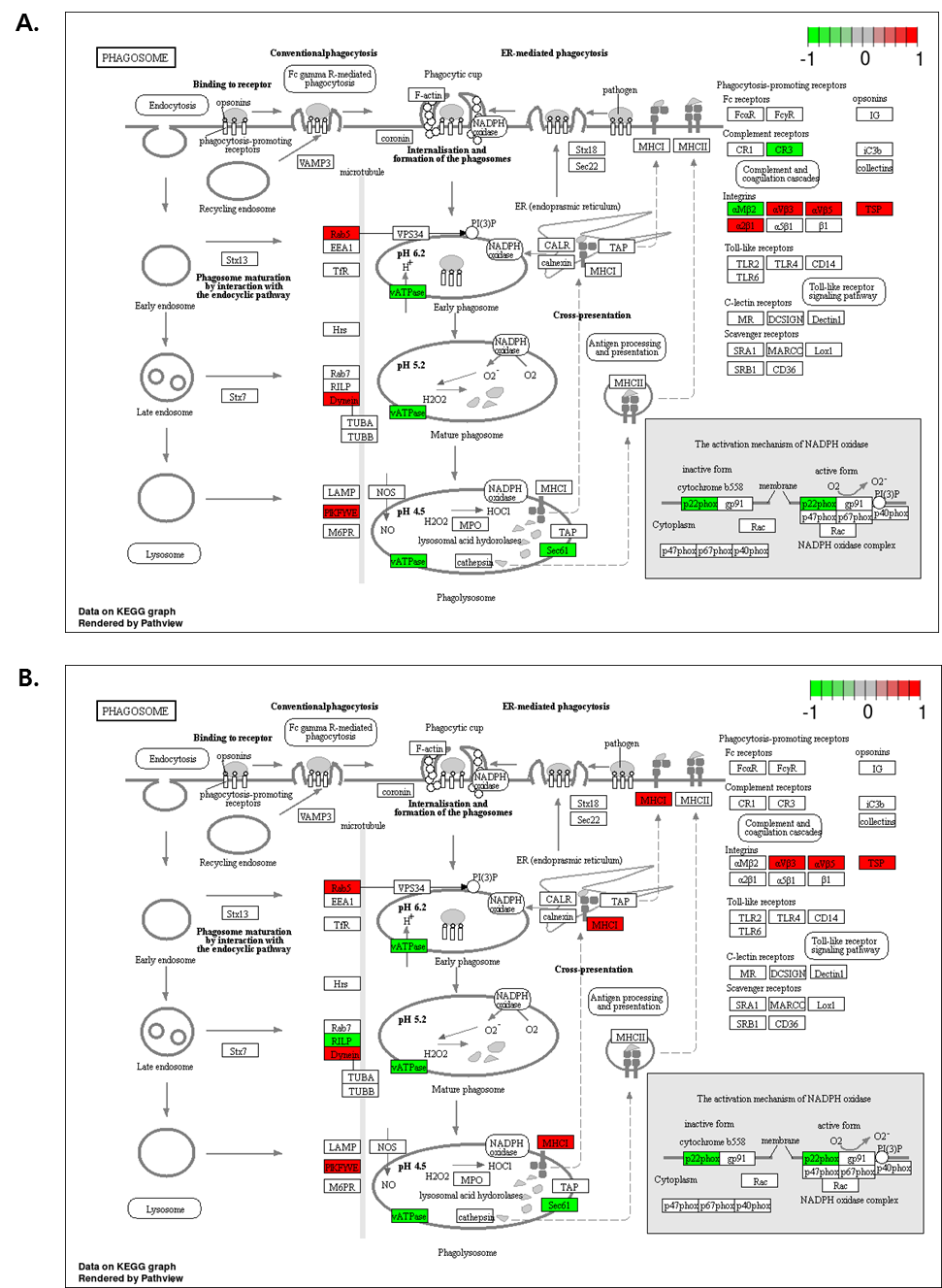


**Supplementary figure 5. The knockdown of RIG-I (found to be upregulated in ZIKV-infected hUCMSCs) led to modulation of ZIKV and host gene expression.**

A549 cells were transfected with control or RIG-I specific siRNA and infected with ZIKV (MOI=1). *ZIKV NS5, RIG-I, MDA5, IFN-b, ISG15, OAS1*, and *OAS2* gene expression levels were determined by qRT-PCR. Expression of target genes was normalized to that of *β-actin*. The qRT-PCR experiments were performed in duplicates and the average of all experiments is shown. **p < 0.05; **p < 0.01; ***p < 0.001*, versus control siRNA-transfected cells.


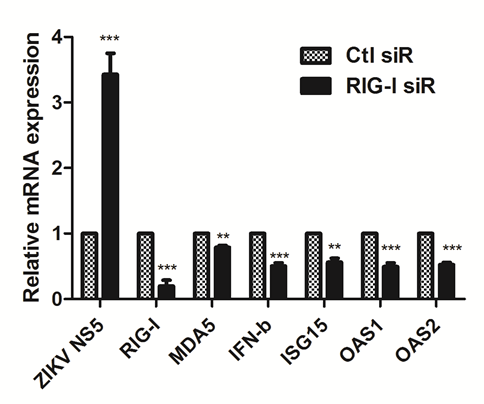


**Supplementary figure S6. ZIKV infection results in alterations in mitochondrial mass in hUCMSCs.**

Confocal microscope analysis of mitochondrial morphology using MitoTracker Green in ZIKV-infected hUCMSCs. ZIKV E protein was immunostained with anti-pan-flavivirus envelope monoclonal antibody. ZIKV E and cell nuclei are stained red and blue, respectively. The images are representative of three independent experiments. Scale bar = 20μM.


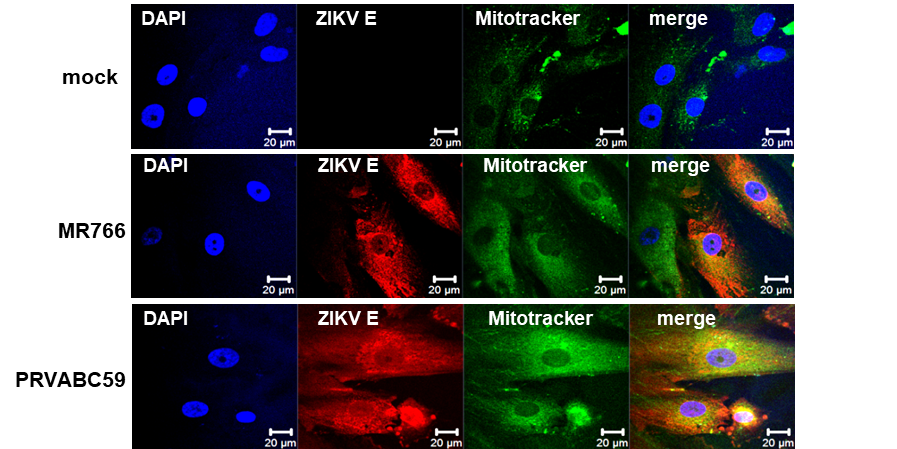


**Supplementary figure S7. ZIKV infection results in increased oxygen consumption rate in hUCMSCs.**

Oxygen consumption rate (OCR) in mock-infected and ZIKV-infected hUCMSCs (MOI=3) was measured in real-time under basal conditions by XF Seahorse analyser. OCR was performed every 7 minutes following ZIKV infection in real time. We measured basal OCR level within 2 hours after ZIKV infection. There was no cytotoxic effect by ZIKV infection at 2 hours, causing no change in cell density. Data represent mean + SD of three biological replicates.


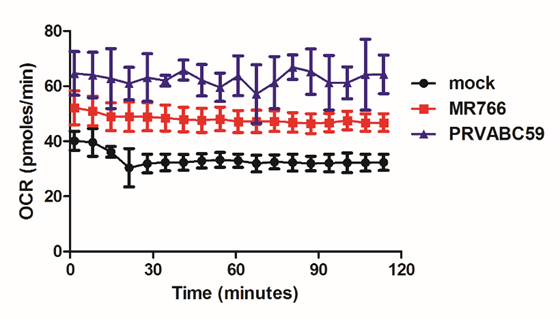


**Supplementary figure S8. Co-localization of ZIKV NS4B with mitochondrial marker TOM20**

Hela cells were co-transfected with empty vector, NS4A-FLAG, NS4B-FLAG, or NS5-FLAG encoding plasmid and TOM20-GFP (mitochondrial marker). Scale bar = 20μM.


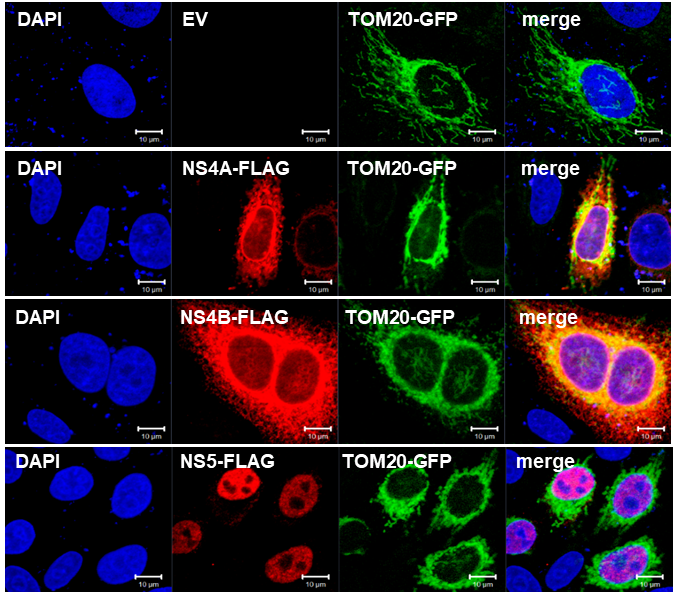


**Supplementary figure S9. ITGAV is important for ZIKV binding to cells**

Cells were transfected with control or *ITGAV*-specific siRNA cells were absorbed with ZIKV MR766 (MOI of 1) for 1 hour at 4°C. Then, cells were moved to 37°C incubator for 1 hour to allow for virus entry. Cells were then treated with acid glycine to inactivate non-internalized virus for 5 min and washed with cold HBSS. Without permeabilization, ZIKV E protein expression was determined by confocal microscopy. Scale bar = 20 μm.


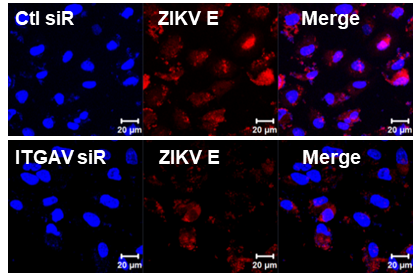


**Supplementary Table 1. Top 20 up-regulated differentially expressed genes (DEGs) in ZIKV-infected hUCMSCs (Innate Immune Function).**

| **RANK** | **MR766, 4 hpi** | | | **MR766, 48 hpi** | | | **PRVABC59, 4 hpi** | | | **PRVABC59, 48 hpi** | | |
| --- | --- | --- | --- | --- | --- | --- | --- | --- | --- | --- | --- | --- |
|  | **Gene Name** | **FC** | ***p-value*** | **Gene Name** | **FC** | ***p-value*** | **Gene Name** | **FC** | ***p-value*** | **Gene Name** | **FC** | ***p-value*** |
| 1 | EIF2AK2 | 2.36 | 0.11885 | IFIH1 | 11.00 | 0.00045 | EIF2AK2 | 1.99 | 0.21215 | CXCL10 | 22.00 | 0.00415 |
| 2 | ADAR | 2.25 | 0.09755 | CXCL10 | 10.70 | 0.00955 | IRF2 | 1.91 | 0.1507 | TNFSF10 | 16.44 | 0.0023 |
| 3 | OAS1 | 2.18 | 0.38065 | IFNB1 | 10.55 | 0.1512 | ADAR | 1.91 | 0.16775 | IFNB1 | 16.11 | 0.1117 |
| 4 | VEGFA | 1.93 | 0.18265 | OAS1 | 10.26 | 0.04365 | MYD88 | 1.64 | 0.25845 | IL6 | 15.13 | 0.0007 |
| 5 | MYD88 | 1.75 | 0.22975 | MX2 | 8.93 | 0.0026 | STAT2 | 1.55 | 0.29665 | OAS1 | 12.81 | 0.00715 |
| 6 | STAT2 | 1.65 | 0.2803 | TNFSF10 | 8.57 | 0.013 | VEGFA | 1.49 | 0.37865 | IFIH1 | 12.81 | 0.0001 |
| 7 | STAT1 | 1.52 | 0.51025 | DDX58 | 7.51 | 0.01025 | PML | 1.25 | 0.6042 | MX2 | 9.91 | 0.000005 |
| 8 | PML | 1.45 | 0.4565 | IL6 | 7.26 | 0.0252 | OAS1 | 1.21 | 0.80615 | CCL2 | 9.00 | 0.00085 |
| 9 | IRF2 | 1.39 | 0.4748 | VEGFA | 6.10 | 0.0065 | STAT1 | 1.11 | 0.86545 | IRF1 | 7.78 | 0.00305 |
| 10 | MX2 | 1.33 | 0.5121 | STAT2 | 5.97 | 0.003 | DDX58 | 1.03 | 0.94045 | DDX58 | 7.62 | 0.00325 |
| 11 | MX1 | 1.33 | 0.5782 | IRF1 | 5.65 | 0.0318 | IFIH1 | 0.92 | 0.8645 | ISG20 | 7.56 | 0.019 |
| 12 | DDX58 | 1.30 | 0.61525 | CCL2 | 5.24 | 0.01345 | MX1 | 0.89 | 0.80855 | VEGFA | 6.40 | 0.0015 |
| 13 | IFIH1 | 1.29 | 0.6032 | EIF2AK2 | 4.95 | 0.02165 | MX2 | 0.81 | 0.63385 | STAT2 | 6.14 | 0.0013 |
| 14 | IRF1 | 0.80 | 0.7008 | STAT1 | 4.69 | 0.1337 | IRF1 | 0.79 | 0.66365 | STAT1 | 4.53 | 0.0475 |
| 15 | IL6 | 0.66 | 0.47455 | MX1 | 4.50 | 0.0444 | TNFSF10 | 0.61 | 0.4682 | MX1 | 4.25 | 0.0272 |
| 16 | TNFSF10 | 0.56 | 0.3694 | ADAR | 4.31 | 0.0152 | CXCL10 | 0.43 | 0.3015 | EIF2AK2 | 4.16 | 0.0158 |
| 17 | CXCL10 | 0.56 | 0.4346 | PML | 4.08 | 0.01345 | CCL2 | 0.36 | 0.0354 | PML | 3.94 | 0.0285 |
| 18 | IRF7 | 0.52 | 0.1936 | IRF2 | 3.94 | 0.05475 | IRF7 | 0.33 | 0.03125 | IRF2 | 3.89 | 0.0288 |
| 19 | CCL2 | 0.41 | 0.08375 | MYD88 | 3.65 | 0.048 | IL6 | 0.25 | 0.03315 | ADAR | 3.81 | 0.018 |
| 20 | ISG20 | 0.35 | 0.12965 | ISG20 | 3.22 | 0.29675 | ISG20 | 0.18 | 0.01145 | MYD88 | 3.50 | 0.02975 |

**Supplementary Table 2. Top 20 down-regulated differentially expressed genes (DEGs) in ZIKV-infected hUCMSCs**

| **RANK** | **MR766, 4 hpi** | | | **MR766, 48 hpi** | | | | **PRVABC59, 4 hpi** | | | **PRVABC59, 48 hpi** | | |
| --- | --- | --- | --- | --- | --- | --- | --- | --- | --- | --- | --- | --- | --- |
|  | **Gene Name** | **FC** | ***p-value*** | **Gene Name** | **FC** | ***p-value*** | **Gene Name** | | **FC** | ***p-value*** | **Gene Name** | **FC** | ***p-value*** |
| 1 | MUC19 | -4.9 | 0.0044 | TMEM191A | -5.69 | 0.0093 | MUC19 | | -5.24 | 0.00445 | IMMP1L | -8.44 | 0.0107 |
| 2 | TLE6 | -4.5 | 0.0349 | KRTAP2-3 | -5.54 | 0.0329 | FTCD | | -4.48 | 0.00775 | DHRS2 | -6.3 | 0.03005 |
| 3 | LINC00520 | -4.09 | 0.047 | DMC1 | -5.39 | 0.0344 | ST8SIA5 | | -4.47 | 0.04655 | MUC19 | -4.69 | 0.0068 |
| 4 | PPP1R1C | -4.06 | 0.01015 | SPTBN4 | -5.06 | 0.00645 | MUC12 | | -4.41 | 0.04575 | CENPM | -4.57 | 5.00E-05 |
| 5 | AP1M2 | -3.46 | 0.0221 | CENPM | -4.96 | 0.00125 | AKR1C3 | | -3.67 | 0.00345 | FOLR3 | -4.41 | 0.04 |
| 6 | CHN2 | -3.38 | 0.0006 | CDCA3 | -4.71 | 0.00125 | TMEM156 | | -3.38 | 0.0186 | CDCA3 | -4.36 | 0.00195 |
| 7 | CTC-454M9.1 | -3.31 | 0.01835 | UBE2C | -4.59 | 0.00045 | TGFBR3L | | -3.32 | 0.022 | SPC24 | -4.3 | 0.04305 |
| 8 | LTK | -3.23 | 0.02665 | TRAIP | -4.57 | 5.00E-05 | CCSER1 | | -3.17 | 0.0116 | CDKN3 | -4.25 | 0.0008 |
| 9 | LIME1 | -2.95 | 0.04565 | DHRS2 | -4.44 | 0.045 | TPRG1 | | -3.12 | 0.01585 | GRIN2A | -4.16 | 0.04895 |
| 10 | COX7A1 | -2.87 | 0.01685 | AURKB | -4.44 | 5.00E-05 | GAL | | -3.11 | 0.0254 | FAM64A | -4.12 | 0.0017 |
| 11 | TMEM160 | -2.81 | 0.0009 | C1QTNF2 | -4.29 | 0.02345 | FHIT | | -3 | 0.0355 | UBE2C | -4.1 | 0.0002 |
| 12 | SHF | -2.7 | 0.0154 | PKMYT1 | -4.24 | 0.00125 | DNASE1L2 | | -2.92 | 0.0393 | PPP1R1C | -4.09 | 0.0151 |
| 13 | C4orf48 | -2.69 | 0.0073 | CDC20 | -4.23 | 0.00045 | NUDT8 | | -2.91 | 0.00945 | KRTAP2-3 | -4 | 0.0033 |
| 14 | CDC42EP5 | -2.56 | 0.00705 | NRGN | -4.21 | 0.04405 | CITED4 | | -2.9 | 0.0018 | ACTC1 | -3.98 | 0.0366 |
| 15 | MIF | -2.51 | 0.0116 | FAM64A | -4.2 | 0.0013 | MAGED4B | | -2.89 | 0.00205 | CDC20 | -3.95 | 0.0004 |
| 16 | LAGE3 | -2.49 | 0.00485 | CDKN3 | -4.18 | 0.0036 | ODF3B | | -2.79 | 0.0226 | TMSB15A | -3.91 | 0.0026 |
| 17 | PRR24 | -2.48 | 0.00395 | NMU | -4.09 | 0.01955 | PRSS36 | | -2.77 | 0.0428 | AURKB | -3.9 | 0.0002 |
| 18 | GADD45GIP1 | -2.47 | 0.0018 | PBK | -4.05 | 0.01675 | COX6A1P2 | | -2.76 | 0.00305 | CENPW | -3.88 | 0.00595 |
| 19 | SCAND1 | -2.41 | 0.0027 | RP11-192H23.4 | -4.02 | 0.00875 | COX7A1 | | -2.75 | 0.01985 | PBK | -3.85 | 0.00945 |
| 20 | TPGS1 | -2.41 | 0.0034 | TMSB15A | -3.98 | 0.0241 | PTGER1 | | -2.71 | 0.01555 | TRIM55 | -3.83 | 0.028 |

**Supplementary Table 3. Top 20 up- vs. down-regulated differentially expressed genes (DEGs) in MR766- vs. PRVABC59-infected hUCMSCs at 48hpi**

| **RANK** | **UP-regulated DEGs** | | | **DOWN-regulated DEGs** | | |
| --- | --- | --- | --- | --- | --- | --- |
|  | **Gene Name** | **FC** | ***p-value*** | **Gene Name** | **FC** | ***p-value*** |
| 1 | RAB11FIP4 | 4.16 | 0.03805 | TNNI3K | -8.73 | 0.02535 |
| 2 | FAM150B | 3.68 | 0.04815 | MUC19 | -5.45 | 0.00195 |
| 3 | GCNT2 | 3.42 | 0.00485 | SLC38A11 | -5.32 | 0.04535 |
| 4 | ZP1 | 3.35 | 0.04445 | SCN5A | -3.64 | 0.029 |
| 5 | ICAM2 | 3.21 | 0.00555 | IGF1 | -3.46 | 0.03585 |
| 6 | NKD1 | 3.02 | 0.03705 | ENPP3 | -3.27 | 0.0265 |
| 7 | ART5 | 2.71 | 0.02435 | AC005307.3 | -3.21 | 0.038 |
| 8 | KCNMB2 | 2.49 | 0.0434 | RP5-1043L13.1 | -3.14 | 0.0034 |
| 9 | ADAP1 | 2.48 | 0.0252 | SCN3A | -3.02 | 0.02725 |
| 10 | SKIDA1 | 2.45 | 0.04755 | NKD2 | -2.98 | 0.03295 |
| 11 | TMEM191A | 2.42 | 0.03215 | TGFA | -2.96 | 0.0249 |
| 12 | BEGAIN | 2.18 | 0.0299 | ITPRIPL1 | -2.86 | 0.029 |
| 13 | RP11-162A12.2 | 2.14 | 0.0389 | DCC | -2.59 | 0.02425 |
| 14 | CNKSR1 | 2.08 | 0.04085 | PLCB2 | -2.58 | 0.04845 |
| 15 | ANKRD65 | 2.01 | 0.04525 | CATSPER2 | -2.57 | 0.0255 |
| 16 | NOL4 | 1.98 | 0.026 | MCMDC2 | -2.4 | 0.0203 |
| 17 | TRAIP | 1.86 | 0.02655 | HIVEP2 | -2.32 | 0.04645 |
| 18 | AKR1C2 | 1.84 | 0.01335 | ASRGL1 | -2.24 | 0.02475 |
| 19 | BATF3 | 1.8 | 0.04545 | PCDHB5 | -2.19 | 0.018 |
| 20 | POPDC3 | 1.79 | 0.0329 | MFAP3L | -2.09 | 0.0105 |

**Supplementary Table 4. Top 10 KEGG pathways enriched in up-regulated differentially expressed genes (DEGs) in ZIKV-infected hUCMSCs.**

|  | **m vs MR766 4hpi** | | | **m vs MR766 48hpi** | | | **m vs PRVABC59 4hpi** | | | **m vs PRVABC59 48hpi** | | |
| --- | --- | --- | --- | --- | --- | --- | --- | --- | --- | --- | --- | --- |
|  | **Name** | **# of overlapping genes** | ***q-value*** | **Name** | **# of overlapping genes** | ***q-value*** | **Name** | **# of overlapping genes** | ***q-value*** | **Name** | **# of overlapping genes** | ***q-value*** |
| **1** | Ribosome | 48/91 | 3.2541 | Cell_cycle | 26/124 | 6.92E-07 | Ribosome | 36/91 | 7.66E-26 | Cell_cycle | 28/124 | 1.17E-08 |
| **2** | Oxidative_phosphorylation | 31/132 | 1.50E-13 | DNA_replication | 11/36 | 4.58E-05 | Huntington's_disease | 32/183 | 2.54E-07 | DNA_replication | 11/36 | 9.34E-05 |
| **3** | Huntington's_disease | 39/183 | 3.34E-12 | Parkinson's_disease | 18/130 | 0.000681299 | Oxidative_phosphorylation | 22/132 | 5.42E-07 | African_trypanosomiasis | 8/35 | 0.022697543 |
| **4** | Parkinson's_disease | 29/130 | 3.83E-12 | Spliceosome | 17/127 | 0.005252576 | Parkinson's_disease | 22/130 | 5.42E-07 | Mismatch_repair | 6/23 | 0.038132564 |
| **5** | Alzheimer's_disease | 35/167 | 5.74E-11 | Oxidative_phosphorylation | 16/132 | 0.005277827 | Alzheimer's_disease | 25/167 | 0.00011511 | Base_excision_repair | 7/33 | 0.038132564 |
| **6** | Cardiac_muscle_contraction | 13/77 | 0.009399663 | Huntington's_disease | 22/183 | 0.009015677 | Pyrimidine_metabolism | 11/99 | 0.222147974 | Malaria | 9/51 | 0.038132564 |
| **7** | Regulation_of_actin_cytoskeleton | 21/213 | 0.274628903 | African_trypanosomiasis | 8/35 | 0.009015677 | Cardiac_muscle_contraction | 9/77 | 0.710418291 | Pyrimidine_metabolism | 13/99 | 0.038132564 |
| **8** | Arrhythmogenic_right_ventricular  _cardiomyopathy_ARVC | 11/74 | 0.274628903 | Pyrimidine_metabolism | 13/99 | 0.023539106 | Axon_guidance | 15/129 | 0.710418291 | Rheumatoid_arthritis | 11/91 | 0.099764657 |
| **9** | RNA_degradation | 9/71 | 0.274628903 | Alzheimer's_disease | 19/167 | 0.033233985 | Proteasome | 5/44 | 0.710418291 | Cytokine-cytokine_receptor_interaction | 23/265 | 0.099764657 |
| **10** | Spliceosome | 12/127 | 0.315156937 | Base_excision_repair | 6/33 | 0.074097501 | Phagosome | 13/153 | 0.710418291 | Parkinson's_disease | 13/130 | 0.099764657 |

**# of overlapping genes: number of DEG in Category / number of Category**

**Supplementary table 5. Sequencing statistics of miRNA profiling in ZIKV-infected hUCMSCs.**

| **Sample** | **Total reads** | **Passing**  **Filters (%)** | **Aligned**  **Reads (%)** | **Precursor**  **miRNA**  **reads** | **Mature**  **miRNA**  **reads** | **Known**  **precursor**  **with >= 5x coverage** | **No.**  **known**  **miRNA** | **No. novel**  **miRNA** |
| --- | --- | --- | --- | --- | --- | --- | --- | --- |
| **hUCMSC_mock** | 13404207 | 99.13 | 90.93 | 14811 | 11795448 | 782 | 492 | 279 |
| **hUCMSC_MR766**  **_4hpi** | 13364106 | 99.27 | 90.83 | 16297 | 8711801 | 766 | 493 | 315 |
| **hUCMSC_MR766**  **_48hpi** | 15429286 | 99.07 | 91.31 | 19047 | 9413509 | 745 | 512 | 355 |
| **hUCMSC_PRVABC59**  **_4hpi** | 13216372 | 88.48 | 19083 | 19083 | 8690786 | 750 | 500 | 314 |
| **hUCMSC_PRVABC59**  **_48hpi** | 13410327 | 98.87 | 89.44 | 16860 | 7711805 | 754 | 493 | 300 |

**References**

1. Kanehisa, M.; Sato, Y.; Furumichi, M.; Morishima, K.; Tanabe, M., New approach for understanding genome variations in KEGG. *Nucleic Acids Res* **2019,** 47, (D1), D590-D595.

2. Kanehisa, M., Toward understanding the origin and evolution of cellular organisms. *Protein Sci* **2019,** 28, (11), 1947-1951.

3. Kanehisa, M.; Goto, S., KEGG: kyoto encyclopedia of genes and genomes. *Nucleic Acids Res* **2000,** 28, (1), 27-30.
